# Multifunctional nanoplatform as nano-inducer of ferroptosis for targeted recognition and imaging-guided therapy of metastatic prostate cancer

**DOI:** 10.1016/j.mtbio.2025.102317

**Published:** 2025-09-18

**Authors:** Liang He, Hao Liang, Jixue Wang, Annan Liu, Lei Li, Ji Lu, Ze Wang, Andrew K. Whittaker, Quan Lin

**Affiliations:** a Department of Urology, The First Hospital of Jilin University, Changchun, 130021, Jilin, China; b State Key Laboratory of Supramolecular Structure and Materials, College of Chemistry, Jilin University, Changchun, 130012, China; c Department of Hand Surgery, The Second Hospital of Jilin University, Changchun, 130041, China; d Joint International Research Laboratory of Ageing Active Strategy and Bionic Health in Northeast Asia of Ministry of Education, Changchun, 130041, China; e Australian Institute for Bioengineering and Nanotechnology, The University of Queensland, Brisbane, QLD, 4072, Australia

**Keywords:** Metastasis prostate cancer, Ferroptosis nano-inducer, Tumor targeting, Nanozyme, Multi-mode imaging

## Abstract

Metastasis prostate cancer (PCa) precision detection and effective treatment remain significant challenge in clinic. Ferroptosis brought promising therapeutic strategy for the treatment of metastatic PCa, effectively inducing ferroptosis in PCa cells represents key to improve therapeutic efficacy. Herein, we developed a multifunctional nanoplatform Fe/Au nanodots-bombesin (FGN-BBN) as the ferroptosis nano-inducer to generate large amount of ROS to induce ferroptosis through an “open-source throttling” strategy for targeted imaging-guided therapy of metastatic PCa. On the one hand, FGN-BBN serves as an efficient biomimetic nanozyme and photothermal agent, exhibiting great POD-like activity and generating abundant reactive oxygen species (ROS) via photothermal-enhanced chemodynamic therapy (CDT) to induce ferroptosis, which is achieving “open source” aspect. On the other hand, FGN-BBN exhibit GPx-like activity that depletes overexpressed glutathione (GSH) within the tumor microenvironment, thereby preventing the neutralization of ROS and achieving the “throttling” effect. Furthermore, bombesin facilitates targeted delivery of the nanozyme to metastatic PCa cells, synergistically enhancing ferroptosis activity. In terms of diagnosis, FGN-BBN possesses targeted recognition capabilities and enables multimode bioimaging including fluorescence (FL), computed tomography (CT), and magnetic resonance imaging (MRI), allowing for the “visualization” of tumor localization and real-time imaging-guided therapy. In summary, the multifunctional nanoplatform integrates multienzyme activity, targeted recognition, multimodal imaging, photothermal therapy, and CDT to induce high-efficiency ferroptosis, offering an effective theranostic strategy for metastatic PCa.

## Graphical abstract

**Figure:**
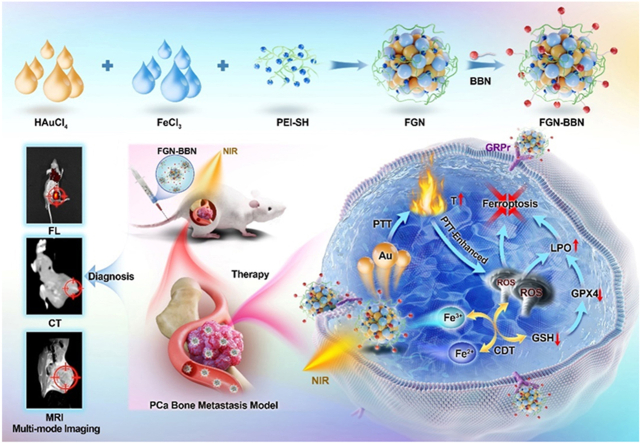

## 1 Introduction

Prostate cancer (PCa) is the most common cancer type among male urogenital system [1]. PCa frequently progresses to castration-resistant prostate cancer (CRPC), which is characterized by poor prognosis and resistance to conventional therapies [2]. Metastatic spread is common in CRPC, with bone being the most frequent and clinically significant site [3]. Accurate diagnosis of primary and metastatic PCa relies on a combination of imaging modalities such as computed tomography (CT), magnetic resonance imaging (MRI), and bone scans [4]. However, these approaches often require multiple imaging sessions, increase diagnostic complexity, and delay therapeutic intervention. Therefore, the development of integrated, multimodal imaging strategies capable of detecting metastatic lesions is urgently needed to improve diagnostic accuracy and overcome the limitations of single-mode detection. From therapeutic perspective, conventional PCa treatment modalities, such as surgery, chemotherapy, and radiotherapy. Surgical resection is often not feasible for advanced or metastatic PCa, while chemotherapy and radiotherapy are hindered by severe side effects and resistance development. [5]. Thus, there is an urgent need for innovative therapeutic strategies for metastatic PCa that improve treatment precision while minimizing systemic toxicity [6].

Ferroptosis is a recently characterized form of programmed cell death that is genetically and biochemically distinct from caspase-dependent apoptosis, pyroptosis, and necrosis. It has emerged as a promising strategy to overcome therapeutic resistance in cancers that are refractory to conventional treatment regimens [7]. Therefore, efficiently inducing ferroptosis in tumor cells is considered critical to enhancing cancer treatment outcomes. Among various strategies, the development of nanomaterials offers a particularly promising approach for inducing ferroptosis [8]. In this context, nanozymes—a class of nanomaterials that mimic natural enzyme activities—have attracted considerable attention. These nanozymes typically incorporate transition metal ions (e.g., Fe^2+^ or Cu^+^), which catalyze Fenton or Fenton-like reactions to produce excessive reactive oxygen species (ROS), thereby triggering lipid peroxidation and ferroptotic cell death in tumor cells. [9,10]. This process forms the foundation of chemodynamic therapy (CDT), a nano-catalytic approach that exploits the elevated H_2_O_2_ levels in the tumor microenvironment to selectively induce oxidative damage while minimizing harm to normal tissues. Furthermore, elevated temperatures have been shown to enhance the catalytic activity of nanozymes, thereby amplifying ROS production and improving the therapeutic efficacy of ferroptosis-based treatments.

To further enhance ferroptosis induction and CDT efficacy, recent efforts have focused on integrating nanozyme activity with photothermal platforms. Among these, gold-based nanomaterials (GNs) have garnered increasing attention due to their remarkable physicochemical properties. Owing to their ultrasmall size, facile surface modification, and outstanding optical and thermodynamic characteristics, GNs not only serve as effective agents for photothermal therapy (PTT), but also act as efficient carriers or co-catalysts in ROS-generating system [[11], [12], [13], [14]]. For therapeutic perspective, GNs exhibit strong near-infrared (NIR) absorption and excellent photothermal conversion efficiency, enabling localized heating for tumor ablation [14,15]. More importantly, GNs-mediated hyperthermia has been shown to amplify the catalytic activity of nanozymes, thereby accelerating ROS production and potentiating ferroptosis-based treatments [[16], [17], [18]]. Moreover, GNs exhibit intrinsic fluorescence (FL) and high X-ray attenuation, making them suitable for multimodal imaging including FL and CT. Meanwhile, Fe-based nanomaterials exhibit superparamagnetic properties, enabling their application in MRI as efficient contrast agents. Based on the above advantages of Fe and Au, the integration of both into a single nanoparticle through a simple and effective preparation method will provide a promising strategy for the diagnosis and treatment of metastatic PCa [6,12].

Despite these advances, the effectiveness of ferroptosis-based strategies remains limited by the intrinsic antioxidant systems within tumor cells, particularly the glutathione (GSH)/glutathione peroxidase 4 (GPX4) axis. GSH, a major intracellular antioxidant, scavenges ROS and prevents lipid peroxidation, thereby inhibiting ferroptosis [19]. GPX4, a selenoenzyme that utilizes GSH to reduce lipid hydroperoxides, is considered a master regulator of ferroptosis [20]. The GPX4 pathway is critical in maintaining cellular redox homeostasis by reducing lipid peroxides, thus preventing ferroptosis. Inhibition of GPX4 or depletion of GSH can promote ferroptosis by accumulating toxic lipid peroxides. This suggests that manipulating GSH/GPX4 pathways could be an effective strategy to sensitize tumors to ferroptosis-based therapies.

In addition to therapeutic potency, target specificity is critical for minimizing systemic toxicity and improving treatment precision. While prostate-specific membrane antigen (PSMA) is a widely used target in PCa, it is not universally expressed—particularly in neuroendocrine or PSMA-negative subtypes [16]. Gastrin-releasing peptide receptor (GRPR) is overexpressed in a broad spectrum of malignancies, including PCa, and serves as a complementary target to PSMA [21,22]. Bombesin (BBN) a GRPR-targeting peptide, has demonstrated excellent potential in tumor imaging and therapy [23]. Incorporating BBN into functional nanoplatforms may therefore enhance tumor targeting, enable GRPR-mediated imaging, and improve therapeutic efficacy.

**Scheme 1:**
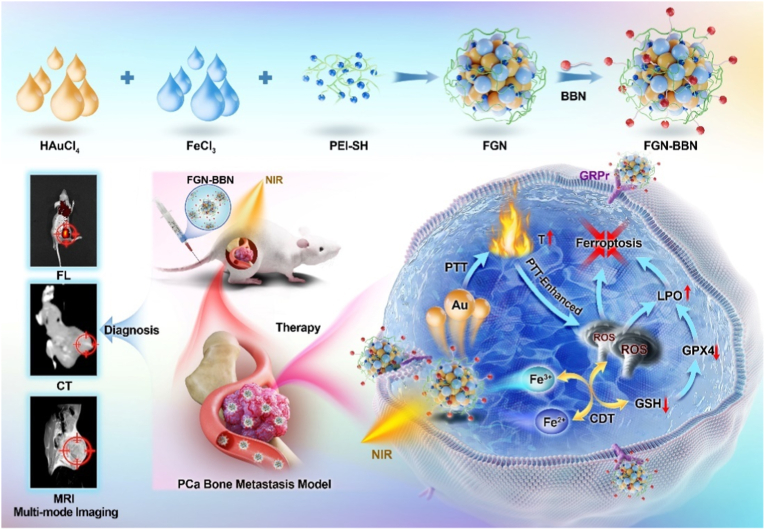
Schematic illustration of the synthesis of FGN-BBN and its application in GRPR-targeted FL/CT/MRI multimodal imaging and combined PTT-Ferroptosis therapy for bone metastatic PCa.

To achieve an integrated theranostic strategy for metastatic PCa, we developed a multifunctional nanoplatform Fe/Au nanodots functionalized with BBN (FGN-BBN), which serving as the ferroptosis nano-inducer to generate high level of ROS to induce ferroptosis through an “Open-source throttling” strategy for targeted imaging-guided therapy (Scheme 1). Initially, Fe/Au nanodots (FGN) were synthesized, exhibiting strong photothermal conversion efficiency and multi-enzyme-like catalytic activity. To impart tumor-targeting capability, BBN was conjugated to the nanodots via amide bond formation, yielding the GRPR-targeted nanosystem FGN-BBN. In terms of treatment, the Fe^2+^ ions in FGN-BBN exhibit peroxidase (POD)-like activity, catalyzing Fenton reaction with overexpressed H_2_O_2_ in tumor cells to produce Fe^3+^ and abundant ROS. The photothermal properties of FGN-BBN further enhance this catalytic activity under NIR, promoting additional ROS generation. Meanwhile, Fe^3+^ ions mimic glutathione peroxidase (GPx)-like activity by consuming intracellular GSH, leading to downregulation of GPX4 and inhibition of ROS scavenging. The regenerated Fe^2+^ then further enhance the Fenton reaction, establishing a self-sustaining catalytic cycle. In addition, FGN-BBN enables targeted recognition and supports multimodal imaging (FL/CT/MRI), allowing real-time tumor localization and precise imaging-guided treatment. Overall, FGN-BBN could integrate GRPR-targeted multimodal imaging with synergistic PTT, CDT and ferroptosis based therapy for the precise diagnosis and treatment of bone-metastatic PCa.

## 2 Methods and materials

### 2.1 Materials

GPX4 antibody (1:500, Servicebio, China), Beta-actin antibody (Servicebio, China), Goat-mouse antibody (Servicebio, China). 2,7-dichlorodihydroffuorescein diacetate (DCFH-DA) was sourced from Servicebio. 4-Hydroxynonenal (4-HNE) antibody (1:500, MedChemExpress, USA). Bombesin peptide (BBN) was purchased from GL Biochem (Shanghai) Ltd. High-glucose Dulbecco's modified Eagle's medium (HG-DMEM) and fetal bovine serum (FBS) were purchased from Gibco (Grand Island, NY, USA). Clear 6-well and 96-well tissue culture polystyrene (TCP) plates were purchased from Corning Costar Co. (Cambridge, MA, USA). Iron chloride hexahydrate (FeCl_3_⋅6H2O), chloroauric acid (HAuCl_4_), N-(3-dimethylaminopropyl)-N′-ethylcarbodiimide hydrochloride (EDC) and N-hydroxysuccinimide (NHS) were purchased from Aladdin; N, Ndimethylformamide (DMF) was purchased from Beijing Chemical Plant; 3-mercaptopropionic acid (MPA) and branched polyethylenimine (PEI, Mw = 600 g/mol) were purchased from Sigma Aldrich; hydrazine hydrate (N_2_H_4_⋅H_2_O), acetone and trichloromethane were purchased from Sinopharm Chemical Reagent Co., LTD. Secondary deionized water was used in the experiment. All reagents were used without further treatment. GRPR positive RM-1 PCa cells and GRPR negative fibroblast NIH-3T3 cells were sourced from our laboratory [12].

### 2.2 Synthesis of ligand (SH-PEI)

A positively charged ligand thiolated polyethylenimine (SH-PEI) was synthesized as follows: N, N dimethylformamide (DMF, 40 mL), 1-ethyl-3-(3-dimethylaminopropyl) carbodiimide hydrochloride (EDC, 64 mg), N-hydroxysuccinimide (NHS, 38 mg) were sequentially added to a three-neck flask and stirred until completely dissolved. Subsequently, 3-mercaptopropionic acid (MPA, 400 μL) was added to the solution and stirred for additional 30 min. Separately, polyethylenimine (PEI, 0.65 g) was dissolved in 2 mL of ethanol, and the resulting solution was added dropwise to the activated reaction mixture under a nitrogen atmosphere. The mixture was then stirred at room temperature for 48 h to ensure complete reaction. After the reaction, the mixture was concentrated using rotary evaporation to a final volume of approximately 3 mL. The concentrate was then washed with 24 mL of acetone followed by 8 mL of trichloromethane. The mixture was centrifuged at 8800 rpm for 15 min to remove the supernatant. The resulting pellet was redispersed in 5 mL of deionized water and stored at −20 °C for subsequent use.

### 2.3 Synthesis of FGN

To synthesize FGN, H_2_O (5 mL), SH-PEI (300 μL), HAuCl_4_ (250 μL, 50 mM), FeCl_3_ (50 μL, 50 mM), and N_2_H_4_⋅H_2_O (300 μL) were sequentially added into a three-necked flask. The whole system was heated in an oil bath at 80 °C for 4 h under constant stirring. After completion, the resulting product was dialyzed against deionized water for 8 h. The purified FGN solution was stored at room temperature in the dark for further use.

### 2.4 Synthesis of FGN-BBN

To synthesize FGN-BBN, 2 mL of the pre-prepared FGN solution was added to a round-bottom flask, followed by the addition of 7.2 mg of EDC and 2.4 mg of NHS. The mixture was stirred at room temperature for 30 min. Subsequently, 80 μL of BBN solution was added into the activated FGN solution and incubated at room temperature for 8 h. The reaction mixture was then transferred into a dialysis bag (molecular weight cut-off: 3500 Da) and dialyzed against deionized water for 12 h to remove excess reagents. The final FGN-BBN product was collected to further characterization.

### 2.5 Characterization of FGN-BBN

The morphology and size of FGN-BBN were characterized using transmission electron microscopy (TEM, JEOL TECNAI F20) operated at 200 kV. Fourier transform infrared (FTIR) spectra were recorded in the range of 500–4000 cm^−1^ using a Nicolet Avatar 360 FTIR spectrophotometer. UV–visible (UV–Vis) absorption spectra were measured by Lambad 800 UV–Vis spectrophotometer. The spectra of proton nuclear magnetic resonance (^1^H NMR) were recorded using a Bruker AVANCEIII 400 NMR spectrometer. The dynamic light scattering (DLS) was measured by Zetasizer Nano ZS particle size analyzer. X-ray photoelectron spectra (XPS) of the samples were measured by VG ESCALAB MKII spectrometer. FL properties were measured by Shimadzu RF-5301 PC FL spectrometer. Additionally, infrared thermal images were captured with an infrared thermal imaging camera (FLIR T420).

### 2.6 Hemocompatibility evaluation of FGN-BBN

The hemocompatibility of FGN and FGN-BBN was evaluated via standard hemolysis assay. 4 % red blood cell (RBC) suspension derived from pig blood was obtained from Zhengzhou Pingrui Biotechnology Co., Ltd. For each test, 1.0 mL of the RBC suspension was added into 1.5 mL centrifuge tubes. Phosphate-buffered saline (PBS) and 1 % Triton X-100 were used as the negative and positive controls, respectively. Samples containing FGN and FGN-BBN at concentrations of 50, 100, 200, and 400 μg/mL were individually added to the tubes. All samples were incubated at 37 °C for 2 h. After incubation, the mixtures were centrifuged at 8000 rpm for 5 min. The supernatants were observed for visible hemolysis and subsequently photographed. Subsequently, 200 μL of each supernatant was transferred into a 96-well plate in triplicate, and the absorbance at 540 nm was measured using a microplate reader (CLARIOstar, BMG Labtech, Germany). The hemolysis ratio was calculated based on the absorbance values of the experimental groups relative to the positive and negative controls.

### 2.7 Biocompatibility assay

The cell biocompatibility was evaluated using a Cell Counting Kit-8 (CCK-8) assay. Briefly, 3T3 cells were seeded in 96-well plates at a density of 5 × 10^3^ cells per well and allowed to adhere for 24 h. Subsequently, various concentrations (50, 100, 200, and 500 μg/mL) of GN, FGN, and FGN-BBN nanoplatforms were dispersed in PBS and added to the respective wells.

After 24h co-cultured, the medium was removed and replaced with 100 μL of fresh medium containing 10 % CCK-8 solution. The cells were then incubated at 37 °C for an additional 2 h. Finally, the optical density (OD) at 450 nm was measured by using a microplate reader (CLARIOstar, BMG Labtech, Germany). Each condition was tested in triplicate, and the mean OD value of the three replicates was calculated for analysis. Finally, microplate reader (BMG CLARIOstar, Germany) was using to test the optical density (OD) value of the sample at 450 nm. Each sample at every concentration was tested in triplicate, and the average OD value of the three replicates was calculated. Cell viability (%) was calculated using the following Formula 1.

Formula 1 $$\text{Cell}\;\text{Viability}\left(\%\right)=\frac{{\text{OD}}_{\text{sample}}-{\text{OD}}_{\text{blank}}}{{\text{OD}}_{\text{control}}-{\text{OD}}_{\text{blank}}}\times 100\%$$

OD_sample_ is the absorbance of treated group, OD_blank_ is OD of the absorbance of blank group, OD_control_ of the control group.

### 2.8 Cellular uptake and targeting evaluation

To quantitatively analysis the GRPR-targeted endocytosis of FGN-BBN, both confocal laser scanning microscopy (CLSM) and flow cytometry (FCM) were employed. For CLSM analysis, GRPR-positive RM-1 cells and GRPR-negative NIH-3T3 cells were seeded at a density of 1 × 10^5^ cells per well in 6-well plates containing sterile cover slips. After 24 h of incubation to allow for cell adhesion, FGN or FGN-BBN (200 μg/mL) was added to the designated wells. At 4- and 12-h post-incubation, the culture medium was removed, and the cells were washed three times with PBS. Cells were then fixed with 4 % paraformaldehyde at room temperature for 20 min, followed by another PBS washes. Subsequently, the cell nuclei were stained with DAPI for 5 min. Then, the cover slips were then carefully removed, mounted onto glass slides, and imaged using CLSM to visualize the cellular internalization of FGN and FGN-BBN.

For FCM analysis, RM-1 and 3T3 cells were seeded in 6-well plates at a density of 2 × 10^5^ cells per well and cultured for 24 h. Then, 200 μg/mL of FGN or FGN-BBN was added to the assigned wells. After incubation for 3 and 12 h, cells were washed three times with PBS, harvested, and subjected to FCM analysis to quantify FL intensity, thereby evaluating the cellular uptake efficiency.

### 2.9 Photothermal-induced cytotoxicity assay of FGN-BBN

RM-1 cells (8 × 10^3^) were seeded into 96-well plates and incubated for 24 h to allow for cell adhesion. Following attachment, 500 μg/mL of FGN or FGN-BBN was added to the designated wells. After 12 h of co-incubation, the cells were exposed to NIR laser irradiation (808 nm, 1.5 W/cm^2^) for 5 min. Post-irradiation, the cells were cultured for an additional 3 h. Subsequently, the medium containing FGN or FGN-BBN was removed and replaced with 100 μL of fresh DMEM supplemented with 10 % CCK-8 solution. After incubation for 2 h at 37 °C, the absorbance was measured at 450 nm using a microplate reader (CLARIOstar, BMG Labtech, Germany). The relative cell viability was calculated using Formula 1.

### 2.10 Live/Dead cell staining assay

RM-1 cells (4 × 10^5^) were seeded in 12-well plates and cultured for 12 h to allow adhesion. After attachment, 500 μg/mL of FGN or FGN-BBN was added to the corresponding wells. The FGN + NIR and FGN-BBN + NIR groups were subsequently exposed to NIR laser irradiation (808 nm, 1.5 W/cm^2^) for 5 min. After 24 h of incubation, cells from each group were harvested, stained using a LIVE/DEAD viability assay kit (Servicebio, China), and immediately imaged using a FL microscope. Semi-quantitative analysis of FL intensity was conducted using ImageJ software (National Institutes of Health, Bethesda, MD, USA).

### 2.11 Wound healing assay of NIR induced FGN-BBN treatment

A wound healing assay was performed to evaluate the migratory ability of PCa cells. RM-1 cells (1 × 10^6^) were seeded in 6-well plates and cultured until reaching approximately 90–100 % confluence. Then, sterile 200 μL pipette tip was used to create a straight scratch across the cell monolayer. The wells were gently washed twice with PBS to remove detached cells. Then, 2 mL fresh serum-free DEME medium was added for each well. The different well was treated with 200 μg/mL FGN and FGN-BBN. After 12h co-cultured, FGN-BBN + NIR group was exposed to NIR light (808 nm, 1.5 W/cm^2^) for 5 min. Images of the scratch area were captured at 0 h, 24 h, and 48h. The wound closure was quantified by measuring the wound area at each time point using ImageJ software, and the migration rate was calculated as the percentage of wound closure relative to the initial wound area.

### 2.12 Intracellular ROS, LPO, and GSH detection after NIR-induced FGN-BBN treatment

Intracellular ROS generation was assessed using a 2′,7′-dichlorodihydro fluorescein diacetate (DCFH-DA) probe (Servicebio, China). RM-1 cells (3 × 10^5^) were seeded into 12-well plates and allowed to adhere for 24h. Subsequently, 500 μg/mL of FGN and FGN-BBN was added to the designated wells. The FGN + NIR and FGN-BBN + NIR groups were exposed to NIR laser irradiation (808 nm, 1.5 W/cm^2^) for 5 min. After 24 h of incubation, cells were stained with DCFH-DA according to the manufacturer's instructions. FL images were acquired using CLSM to evaluate ROS levels.

Lipid peroxidation (LPO) was quantified using a malondialdehyde (MDA) assay kit (Service bio, China). RM-1 cells (8 × 10^5^) were seeded in 6-well plates and cultured for 12 h. After discarding the old medium, 2 mL of fresh culture medium containing 500 μg/mL FGN or FGN-BBN was added. Then, cells in FGN + NIR and FGN-BBN + NIR group were exposed to NIR (808 nm, 1.5 W/cm^2^) for 5 min. After additional 2-h incubation, the cells were harvested and processed using the MDA assay kit following the manufacturer's instructions. Absorbance was measured at 532 nm, and MDA levels were expressed as fold change relative to the control group.

Furthermore, LPO was further assessed using BODIPY™ 581/591C11 staining. RM-1 cells (3 × 10^5^) were seeded into 6-well plates containing sterile glass coverslips. After 12 h of incubation, 500 μg/mL of FGN or FGN-BBN was added. After 6 h of treatment, the FGN + NIR and FGN-BBN + NIR groups were irradiated with NIR (808 nm, 1.5 W/cm^2^) for 5 min. Then, 1.0 μM BODIPY C11 was added to each well and incubated at 37 °C in the dark for 30 min. After washing three times with PBS, the coverslips were mounted and imaged using CLSM.

Intracellular GSH levels were measured using a total GSH assay kit (Servicebio, China). RM-1 cells (8 × 10^5^) were seeded in 6-well plates and cultured for 12 h. Cells were then treated with 500 μg/mL FGN or FGN-BBN in 2 mL of fresh medium. The FGN + NIR and FGN-BBN + NIR groups were subjected to NIR irradiation (808 nm, 1.5 W/cm^2^) for 5 min. After treatment, cells were lysed, and total GSH levels were quantified according to the kit protocol.

### 2.13 Ferroptosis inhibition assay using Ferrostatin-1(Fer-1)

A ferroptosis rescue experiment was performed to verify the ferroptosis-inducing capability of the FGN-BBN. RM-1 cells (8 × 10^5^) were seeded into 96-well plates and allowed to adhere overnight. Ferrostatin-1 (Fer-1, 0.1 μM, dissolved in 1 μL DMSO) was added for pretreatment to inhibit ferroptosis. After 24 h, the medium was removed and replaced with 200 μL of DMEM containing FGN or FGN-BBN. Each group included five replicate wells.

For the FGN + NIR and FGN-BBN + NIR groups, cells were irradiated with NIR laser (808 nm, 1.5 W/cm^2^) for 5 min, followed by an additional 12-h incubation. Subsequently, the treatment medium was discarded and replaced with 100 μL of fresh DMEM supplemented with 10 % CCK-8 reagent. After incubation at 37 °C for 2 h, the absorbance at 450 nm was measured using microplate reader (CLARIOstar, BMG Labtech, Germany) to assess cell viability. The relative cell viability was calculated using Formula 1**.**

### 2.14 Western blot analysis of GPX4 expression

Western blot analysis was performed to examine the expression of the ferroptosis-related protein GPX4. RM-1 cells were treated under the same conditions as described in the intracellular GSH detection experiment. Total cellular proteins from each group were extracted using RIPA lysis buffer (Beyotime, China), and protein concentrations were quantified using a BCA protein assay kit (Beyotime, China).

Then, Equal amounts of protein were loaded for SDS-PAGE electrophoresis, performed at 80 V for 30 min followed by 120 V for 50 min. The proteins were then transferred onto PVDF membranes at 300 mA for 30 min. Membranes were blocked with 5 % skim milk in TBST for 2 h at room temperature, then incubated overnight at 4 °C with primary antibodies against GPX4. After washing, membranes were incubated with HRP-conjugated secondary antibodies for 2 h at room temperature. The protein bands were visualized using a chemiluminescence imaging system (Servicebio, China), and densitometric analysis was performed using ImageJ software (National Institutes of Health, Bethesda, MD, USA).

### 2.15 Establishment of PCa bone metastasis model

PCa bone metastasis model followed the protocol based on our previous reported protocol [12]. Six-week-old male C57BL/6 mice and BALB/c nude mice (weighing approximately 20 g) were employed in this work. BALB/c nude mice were obtained from Beijing Sibeifu Biotechnology Co., Ltd., and C57BL/6 mice were purchased from Liaoning Changsheng Biotechnology Co., Ltd. All animal experiments were performed in accordance with Animal Care and Use Committee of Jilin University Basic Medical College. Mice were anesthetized with isoflurane, and 1 × 10^6^ RM-1 PCa cells suspended in PBS were injected into the right tibial cavity of both C57BL/6 and BALB/c nude mice. Following tumor cell inoculation, tumor progression was monitored daily, and tumor volume was calculated using Formula 2.

Formula 2 $$V\left({\text{mm}}^{3}\right)=\frac{1}{2}\times \text{Length}\times {\text{Width}}^{2}$$

V represents tumor volumes, Length represents longest diameter, and Width represents shortest diameter.

### 2.16 In vivo GRPR-targeted multimodal imaging via FL, CT, and MRI

Male BALB/c nude mice bearing PCa bone metastasis tumors were used for FL imaging, while male C57BL/6 mice with established bone metastases were selected for CT and MRI analysis. Once tumor volumes reached approximately 300 mm^3^, mice were intravenously injected with FGN or FGN-BBN at a dose of 5.0 mL kg^−1^ body weight. At predetermined time points post-injection, mice were anesthetized using isoflurane. In vivo FL imaging was performed using the PerkinElmer IVIS Lumina LT III system. CT and MRI scans were conducted using a clinical CT scanner (SOMATOM Sensation 64, Siemens, Germany) and a clinical MRI system (Ingenia, PHILIPS, Netherlands), respectively. For FL imaging, the PerkinElmer IVIS Lumina LT III system was used to acquire images. Tumor regions were selected as regions of interest (ROI), and fluorescence intensity values were obtained using the system's analysis software. For CT, the images were imported into a free DICOM viewer. Quantitative analysis was performed by extracting Hounsfield Unit (HU) values from the tumor regions. For MRI analysis, T1-weighted images were first imported into DICOM viewer, and semi-quantitative analysis was performed using ImageJ software by measuring the mean grayscale values within defined tumor regions to calculate the relative MRI intensity.

### 2.17 In vivo photothermal imaging

Male C57BL/6 mice bearing PCa bone metastasis tumors (∼300 mm^3^) were selected for in vivo photothermal imaging. Mice were intravenously injected with 100 μL of either PBS or FGN-BBN solution at a dosage of 5.0 mL kg^−1^ body weight. Three hours post-injection, the mice were anesthetized and exposed to an 808 nm NIR laser at a power density of 1.5 W/cm^2^ for 5 min. During irradiation, thermal images were captured, and the temperature changes at the tumor sites were recorded using an infrared thermal imaging system.

### 2.18 Therapeutic evaluation of FGN-BBN-induced photothermal and ferroptosis effects in PCa bone metastases

Male C57BL/6 mice bearing RM-1 bone metastasis xenografts were employed for the in vivo therapeutic evaluation. Once the tumor volume reached approximately 100 mm^3^, the mice were randomly assigned into five groups (n = 5 per group): Control (Ctrl), FGN, FGN-BBN, FGN + NIR, and FGN-BBN + NIR. For the FGN, FGN-BBN, FGN + NIR, and FGN-BBN + NIR groups, mice received intravenous injections of FGN or FGN-BBN at a dose of 5.0 mL kg^−1^ body weight every two days. For the PTT treatment groups (FGN + NIR and FGN-BBN + NIR), tumors were exposed to an 808 nm NIR laser at a power density of 2.0 W/cm^2^ for 5 min, administered 2 h post-injection. Tumor volume and body weight were measured everyday throughout the treatment period. After four treatment cycles, the mice were euthanized by cervical dislocation, tumors and major organs were harvested for subsequent analyses.

### 2.19 Histological evaluation of FGN-BBN efficacy and safety

After the in vivo antitumor experiment, the tumor and major organs (i.e., the heart, liver, spleen, lung, and kidney) were harvested and fixed in 4 % (w/v) paraformaldehyde in PBS at room temperature for 24h. Subsequently, the samples of tumor and major organs were treated by dehydration, clearing, wax infiltration, and embedding. Finally, the H&E-stained sections were observed under microscope.

For immunohistochemical (IHC) and immunofluorescence staining, Paraffin-embedded tumor tissue sections each with a thickness of ∼5 μm were prepared. The Ki-67, Caspase-3, TUNEL, GPX4, and 4-HNE activities were detected according to the routine method: (1). Antigen retrieval, (2). Serum closing, (3). Primary antibody addition, and (4). Second antibody addition. To quantitively evaluate TUNEL expressions, tumor sections from each group were imaged under a fluorescence microscope. Five random fields of view per slide were selected, and the fluorescence intensity was analyzed using ImageJ software to generate semi-quantitative results. For Caspase-3, Ki-67, GPX4, and 4-HNE expression, five random fields of view were selected per tumor section, and the number of positively stained cells in each field was counted. The average number of positive cells was calculated and used as the semi-quantitative expression level, analyzed using ImageJ software.

### 2.20 Statistical analysis

All experiments in this study were performed in triplicate. Data are presented as the mean ± standard deviation (SD). Statistical analyses were conducted using SPSS 13.0 software (SPSS Inc., Chicago, IL, USA). Student's t-test was used to evaluate significance. A p-value of <0.05 was considered statistically significant, while p < 0.01 and p < 0.001 were regarded as highly significant.

## 3 Results and discussion

### 3.1 Synthesis strategy and characterization of FGN-BBN

First, a positively charged and multi-thiol-functionalized polymer, SH-PEI, was synthesized by conjugating the carboxyl groups of 3-mercaptopropionic acid (MPA) to the amine groups of polyethyleneimine (PEI) via an amide condensation reaction. Then, SH-PEI was employed as stabilizing and functional ligand for the synthesis of FGN through a co-reduction approach. FGN nanodots demonstrated excellent photothermal conversion efficiency, catalytic activity, and multimodal imaging capabilities. To enable tumor-targeting capability, GRPR targeting peptide BBN was covalently attached to FGN via an amide reaction, forming the targeted nanoplatform FGN-BBN.

**Fig. 1:**
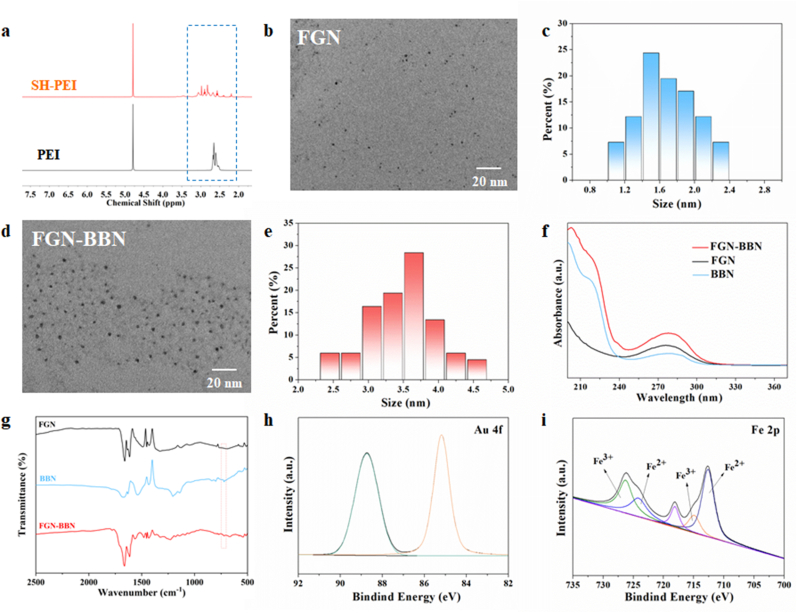
Structural, morphological, and compositional characterization of FGN-BBN. (a) ^1^H NMR spectrum of SH-PEI and PEI. (b) TEM image and (c) size distribution histogram of the FGN. Scale bar: 20 nm (d) TEM image and (e) size distribution histogram of the FGN-BBN. Scale bar: 20 nm (f) UV–vis absorption spectrum of FGN-BBN, FGN and BBN. (g) FT-IR spectra of FGN, BBN and FGN-BBN. High-resolution XPS spectra of (h) Au 4f and (i) Fe 2p of FGN-BBN.

The successful synthesis of SH-PEI was confirmed by ^1^H NMR spectroscopy, which revealed two characteristic triplet peaks in the range of 2.0–3.5 ppm corresponding to the (-CH_2_-CH_2_-) protons of MPA. These peaks were absent in pure PEI, confirming the successful conjugation of MPA to PEI (Fig. 1a). SH-PEI was chosen as the ligand for FGN for two main reasons: (1) the terminal sulfhydryl groups of the ligand coordinate with surface Au(I) on FGN to form Au(I)–thiolate complexes, which is considered to play a key role in the strong FL emission of FGN; and (2) when the thiol rivet of the ligand is on the surface of the FGN, the amine end of the ligand faces outwards, which also provides the possibility for achieving the connection of targeting peptides and proteins through amidation.

The morphology and structure of the resulting nanomaterials were then systematically characterized. Transmission electron microscopy (TEM) images exhibited that FGN possessed a uniform spherical morphology with an average diameter of 1.7 nm (Fig. 1b and c), while FGN-BBN retained a similarly spherical shape with an increased average diameter of 3.5 nm (Fig. 1d and e), indicative of successful surface modification. To further confirm BBN conjugation, UV–Vis absorption spectroscopy, Fouriertransform infrared (FTIR) spectroscopy, and zeta potential measurements were conducted. In the UV–Vis absorption spectrum, a characteristic peak associated with metal−sulfhydryl binding was observed at approximately 273 nm (Fig. 1f). BBN alone exhibited a UV absorption peak at 279 nm, and after conjugation, FGN-BBN showed an absorption peak at 275 nm with significantly increased intensity, confirming the successful attachment of BBN to FGN. FTIR spectra provided additional evidence. The absorption peak at ∼750 cm^−1^, corresponding to the benzene ring of BBN, was absent in FGN but clearly present in both BBN and FGN-BBN (Fig. 1g). This result further confirming successful conjugation of BBN and FGN. Zeta potential analysis (Fig. S1) revealed that FGN had a positive surface charge of +8.42 mV due to the presence of SH-PEI ligand. After BBN conjugation, the surface charge of FGN-BBN increased to +13.64 mV, which may enhance its electrostatic interaction with negatively charged phospholipid bilayers on the cell membrane, thereby facilitating cellular uptake.

To gain further insight into the elemental composition and valence states of FGN-BBN, XPS analysis was performed. The XPS spectra of FGN-BBN showed the presence of both Au 4f and Fe 2p signals (Fig. 1h and i), confirming the coexistence of both Au and Fe elements. The Au 4f spectrum exhibited peaks at 85.2 eV and 88.75 eV, corresponding to Au(I) and Au(0), respectively (Fig. 1h). Au(I) is involved in covalent bonding with the metal-sulfhydryl covalent bonds (Au–S) of SH-PEI, while Au(0) contributes to nanodot nucleation. The Fe 2p spectrum exhibited characteristic binding energies at 715.0 eV and 726.3 eV for Fe(III), and at 712.3 eV and 724.3 eV for Fe(II), confirming the coexistence of both Fe^2+^ and Fe^3+^ in FGN-BBN nanoplatform (Fig. 1i). Additionally, a peak at 163.3 eV in the S 2p spectrum confirmed the presence of sulfur from the thiol groups (Fig. S2). Collectively, these results verify the successful synthesis of FGN-BBN.

### 3.2 Photothermal properties of FGN-BBN

PTT is an emerging treatment modality that utilizes photothermal agents to convert NIR light into heat, thereby inducing localized thermal ablation of tumor tissues. Among various photothermal agents, gold-based nanomaterials have attracted significant attention due to their outstanding photothermal conversion efficiency, excellent biocompatibility, and favorable physicochemical properties [24].

**Fig. 2:**
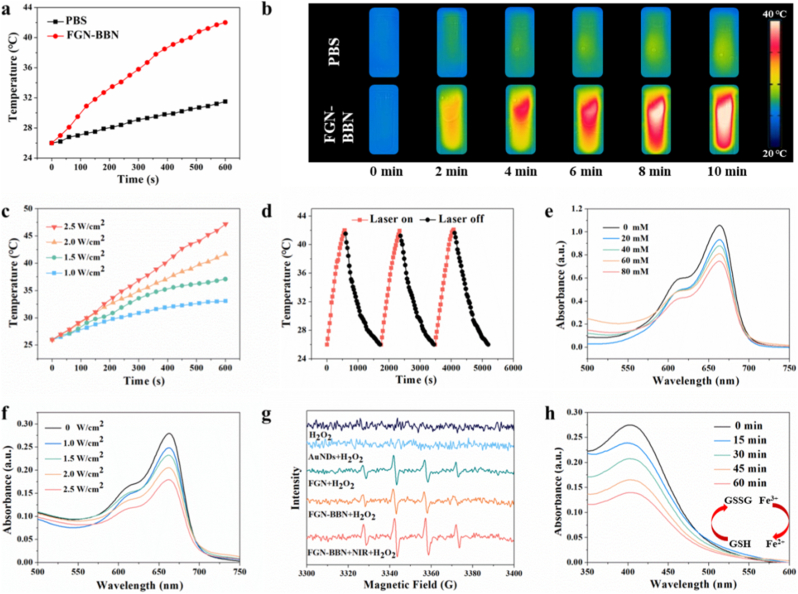
Evaluation of the photothermal performance and catalytic activity of FGN-BBN. (a) Temperature elevation curves and (b) infrared thermal images of PBS (control) and FGN-BBN under 808 nm laser irradiation (2.0 W/cm^2^) over time. (c) Temperature profiles of FGN-BBN under 808 nm laser irradiation at varying power densities (1.0, 1.5, 2.0, and 2.5 W/cm^2^). (d) Photothermal stability of FGN-BBN assessed over three on/off laser irradiation cycles. (e) UV–Vis absorbance spectra of MB after reaction with FGN-BBN and different concentrations of H_2_O_2_. (f) Absorbance of MB after treatment with FGN-BBN under 808 nm laser irradiation at varying power densities. (g) ESR spectra under various reaction conditions with DMPO as a spin trap agent. (h) GSH depletion by FGN-BBN.

In this study, the photothermal capability of FGN-BBN was systematically evaluated. As shown in the photothermal heating curves (Fig. 2a), the temperature of the FGN-BBN solution increased significantly from 26 °C to 41.7 °C upon 10 min of irradiation with an 808 nm laser at a power density of 2.0 W/cm^2^. In contrast, the PBS solution exhibited only a negligible temperature rise under same conditions. This disparity in thermal response was further visualized using infrared thermal imaging (Fig. 2b), indicating the effective photothermal conversion ability of FGN-BBN. In addition, we also tested the temperature rise of FGN-BBN at different power densities. To further explore the relationship between laser power and temperature elevation, FGN-BBN solutions were irradiated with an 808 nm laser at varying power densities ranging from 1.0 to 2.5 W/cm^2^ for 10 min. As shown in Fig. 2c, a laser power-dependent temperature increase was observed, confirming that the photothermal effect of FGN-BBN is positively correlated with laser intensity. In addition, the photothermal stability of FGN-BBN was evaluated through three consecutive cycles of laser irradiation and cooling (Fig. 2d). The temperature of the FGN-BBN remained relatively unchanged across cycles, indicating excellent photothermal stability and reproducibility.

Overall, these findings demonstrate that FGN-BBN possesses robust and stable photothermal properties, demonstrating its promising potential as an effective photothermal agent for tumor.

### 3.3 Enhanced CDT performance of FGN-BBN

CDT based on Fenton reaction is an emerging nanocatalytic therapeutic strategy that converts endogenous H_2_O_2_ into ROS, thereby inducing oxidative stress in cancer cells [25]. Moreover, elevated temperatures are known to accelerate Fenton reaction kinetics, suggesting that the combination of PTT and CDT may offer a promising synergistic approach for enhanced therapeutic efficacy [26]. In this study, the POD-like catalytic activity of FGN-BBN nanoplatform was evaluated using methylene blue (MB) probe to monitor ⋅OH generation via Fenton-like reaction. We first investigated the ability of FGN-BBN to catalyze ⋅OH production in the presence of varying concentrations of H_2_O_2_. As shown in Fig. 2e, the absorbance of MB decreased progressively with increasing H_2_O_2_ concentration, indicating enhanced ⋅OH generation in a concentration-dependent relationship with H_2_O_2_. These results confirm the efficient catalytic performance of FGN-BBN in mediating Fenton reaction.

We further evaluated the effect of pH on the catalytic efficiency of the Fenton reaction. As shown in Fig. S3, MB absorbance decreased more significantly under acidic conditions, indicating that low pH environment promotes the generation of ⋅OH radicals. These findings demonstrate that FGN-BBN can effectively induce the formation of ⋅OH by decomposing H_2_O_2_ in acidic environments.

Importantly, the efficiency of Fenton's reaction is closely related to the reaction temperature [27]. Given the excellent photothermal properties of FGN-BBN, we explored the potential of near-infrared laser mediated photo-assisted Fenton reaction. Accordingly, the catalytic activity of FGN-BBN was evaluated at different laser power densities (1.0, 1.5, 2.0, and 2.5 W/cm^2^) in the presence of H_2_O_2_. As shown in Fig. 2f, increasing the power density resulted in significant reduction in MB absorbance, indicating elevated ⋅OH production under photothermal stimulation.

To further verify ⋅OH generation, 5,5 ′-dimethylpyrrolin-1 oxide (DMPO) was used as a spin-trapping agent for electron spin resonance (ESR) analysis. As shown in Fig. 2g, both FGN and FGN-BBN groups exhibited comparable ESR signals, indicating that conjugation with BBN does not compromise the catalytic activity of FGN. Notably, under 808 nm laser irradiation, the FGN-BBN group displayed significantly enhanced ESR signal intensity, confirming that localized hyperthermia enhances its catalytic efficiency. These results indicate that FGN-BBN enables effective PTT-enhanced CDT by promoting ⋅OH production under NIR irradiation.

In addition to ROS generation, depletion of intratumoral GSH can further amplify the therapeutic effect of CDT [19]. GSH scavenges ROS, thereby reducing CDT efficiency. XPS spectrogram analysis revealed the presence of Fe^3+^ in FGN-BBN, indicating its potential GPx-like activity to consume GSH. To validate this, we assessed GSH depletion by using 5,5′-dithiobis-(2-nitrobenzoic acid) (DTNB). As shown in Fig. 2h, GSH levels declined progressively over time in the presence of FGN-BBN, confirming its GSH-consuming capacity. In conclusion, FGN-BBN demonstrates the ability to react with excessive GSH in the tumor microenvironment, generating Fe^2+^ and oxidized glutathione (GSSG). This not only inhibits ROS clearance but also facilitates continuous Fenton reaction cycles via regenerated Fe^2+^, thereby enhancing CDT efficacy.

### 3.4 In vitro FL, CT and MR imaging performance

Currently, FL, CT and MRI are widely employed in clinical diagnosis. FL imaging is intuitive and sensitive, but suffers from limited spatial resolution. CT imaging offers high spatial resolution, rapid acquisition, and low cost, yet its insufficient sensitivity restricts its effectiveness in soft tissue imaging. In addition, MRI is a non-invasive medical imaging modality with high sensitivity and excellent penetration depth, capable of providing detailed multi-parametric anatomical information of soft tissue. However, MRI is limited in detecting structural lesions in bone [12].

**Fig. 3:**
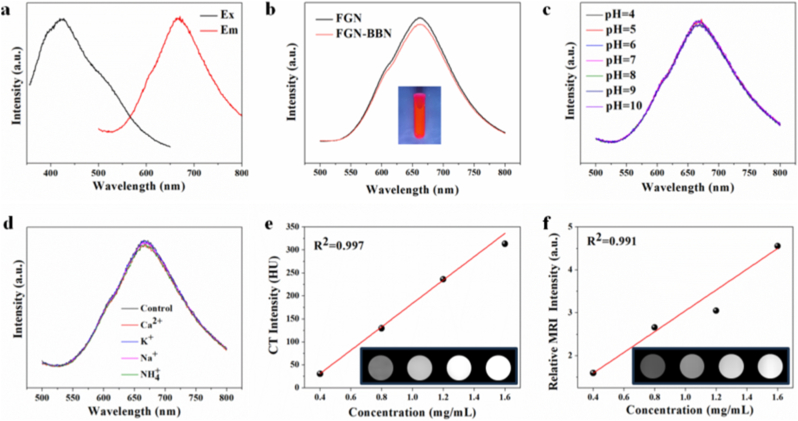
In vitro FL, CT and MR imaging properties of FGN-BBN. (a) Excitation and emission spectra of FGN-BBN. (b) Emission spectra of FGN and FGN-BBN. Inset, photographs of FGN-BBN solution under UV lamp. (c) FL intensity of FGN-BBN at different pH values. (d) FL stability of FGN-BBN in the presence of high concentrations (200 mM) of interfering ions (K^+^, Na^+^, NH_4_^+^, and Ca^2+^). (e) CT signal intensity of FGN-BBN at varying concentrations. Inset: corresponding CT images. (f) Relative MR signal intensity of FGN-BBN at different concentrations. Inset: corresponding T1-weighted MR images.

To overcome the limitations of single imaging modalities, we developed a nanoplatform with integrated multimodal imaging capabilities. We first characterized the FL properties of FGN-BBN. As shown in Fig. 3a, FGN exhibited a strong red FL emission peak at 670 nm under 420 nm excitation. Importantly, after BBN conjugation, FGN-BBN retained its excellent FL characteristics (Fig. 3b). FL stability is a critical factor for in vivo imaging applications. Thus, we evaluated the FL stability of FGN-BBN under physiological pH range (pH 4–10) and common cations (Ca^2+^, K^+^, Mg^2+^, Na^+^). As shown in Fig. 3c and d, FGN-BBN maintained consistent FL intensity under all tested conditions. Moreover, the FL remained stable after storage for 30 days (Fig. S4), demonstrating excellent long-term stability. These results indicate that FGN-BBN exhibits enhanced FL intensity and excellent stability, demonstrating its potential as a promising fluorescent imaging probe for clinical diagnostic applications.

Given the strong X-ray attenuation coefficient of gold, we subsequently evaluated the in vitro CT imaging performance of FGN-BBN to assess its potential as a clinical contrast agent. As shown in Fig. 3e, CT signal intensity increased linearly with FGN-BBN concentration, with a high correlation coefficient (R^2^ = 0.997), indicating that FGN-BBN enables reliable, concentration-dependent CT imaging and may allow for quantitative in vivo tracking.

Furthermore, we also evaluated MRI contrast performance of FGN-BBN. As shown in Fig. 3f, T1-weighted MR signal intensity also increased proportionally with increasing FGN-BBN concentration, with excellent linearity (R^2^ = 0.991), confirming its MR imaging potential. In summary, the synthesized FGN-BBN nanoplatform demonstrates excellent multimodal imaging performance (FL/CT/MRI) in vitro and holds strong potential for future applications in accurate disease diagnosis and image-guided therapy.

### 3.5 In vitro and in vivo GRPR targeting specificity assays of FGN-BBN

Accurate diagnosis remains a clinical challenge in PCa, particularly for PCa with metastatic lesions. GRPR, which is overexpressed on the surface of PCa, plays an important role in PCa detection. Additionally, GRPR can serve as a complementary biomarker to PSMA, enabling the identification of PSMA-negative PCa [28]. BBN, a peptide with high affinity for GRPR, has been widely employed as a GRPR-targeting ligand in both diagnostic and therapeutic applications [29].

Its integration into multifunctional nanostructures, such as FGN-BBN, represents a promising strategy to enhance the specificity and efficacy of targeted treatments [28,30]. Inspired by this, the FGN-BBN nano-system utilize the targeting capability of BBN, exerting a targeted effect on GRPR, thereby enabling the specific tracking of GRPR-positive tumors.

**Fig. 4:**
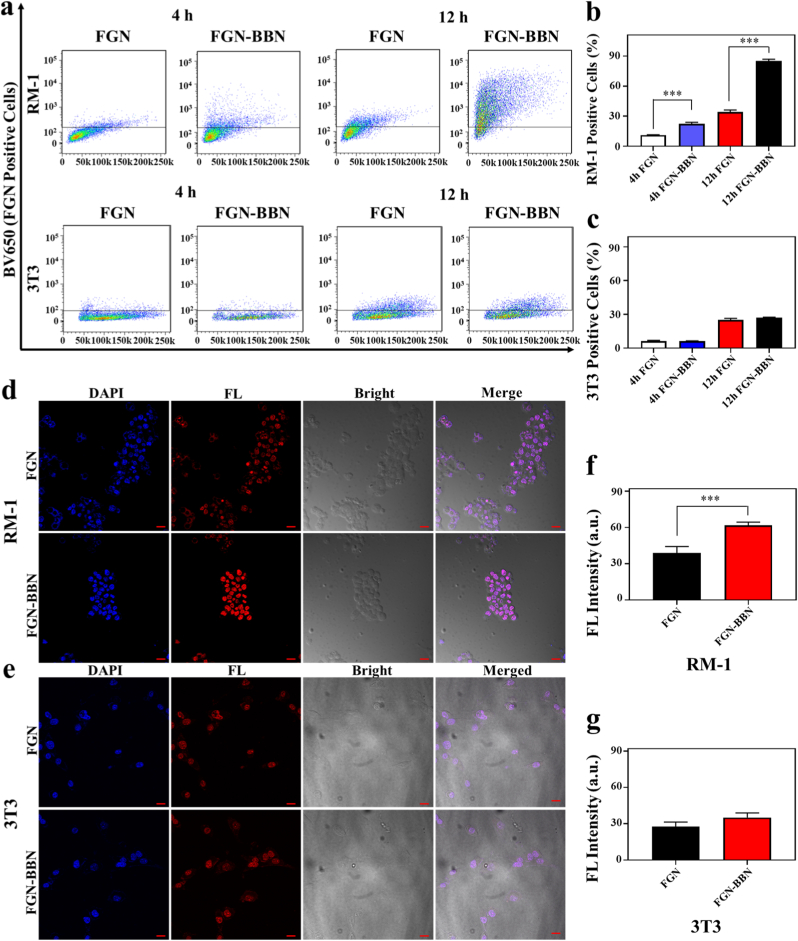
In vitro GRPR targeting specificity assays of FGN-BBN. (a). FCM analysis of cellular uptake of FGN and FGN-BBN in RM-1 and 3T3 cells at 4 h and 12 h. (b). Quantification analysis of FCM results for RM-1 cells. (c). Quantification analysis of FCM results for 3T3 cells. (d) CLSM visualization of FGN and FGN-BBN uptake in RM-1 cells at 12 h. Scale bar: 100 μm. (e) CLSM visualization of FGN and FGN-BBN uptake in 3T3 cells at 12 h. Scale bar: 100 μm. (f). Semi-quantitative analysis of CLSM results in RM-1 cells. (g). Semi-quantitative analysis of CLSM results in 3T3 cells. ∗∗∗p < 0.001.

The mouse PCa cell line RM-1 has been confirmed to exhibit overexpression of GRPR on the cell membrane surface [3]. In contrast, mouse embryonic fibroblasts NIH-3T3 cells with low GRPR expression were selected as the negative control [31]. To assess the GRPR-targeting capability of FGN-BBN, both FCM and CLSM were employed (Fig. 4). First, FCM was used to evaluate the uptake of FGN and FGN-BBN in RM-1 and 3T3 cells (Fig. 4a). Following 4 h of RM-1 cells exhibited significantly stronger FL in the FGN-BBN group, suggesting enhanced cellular uptake via GRPR-mediated targeting. However, no significant difference was observed in 3T3 cells, indicating limited nonspecific uptake in non-tumor cells. After 12 h co-incubation, the proportion of FL-positive RM-1 cells increased markedly in the FGN-BBN group compared to the FGN group (85.06 % vs. 36.4 %, p < 0.001, Fig. 4b), indicating enhanced cellular uptake via GRPR-mediated endocytosis. In contrast, 3T3 cells co-cultured with FGN-BBN for 12 h showed no significant difference in FL-positive cell proportions compared to FGN-treated cells (27.0 % vs. 25.1 %, p > 0.05, Fig. 4c), suggesting minimal nonspecific uptake. Consistent with the FCM results, CLSM results provided visual confirmation of intracellular FL intensity. RM-1 cells treated with FGN-BBN exhibited notably stronger FL signals than those treated with FGN alone after 12 h of co-incubation (Fig. 4d and e). Semi-quantitative analysis of 10.13039/501100007874CLSM images further supported this observation (61.7 vs. 38.9, p < 0.001, Fig. 4f). In contrast, no significant difference in semi-quantitative analysis was observed between the FGN and FGN-BBN groups in 3T3 cells (Fig. 4g).

Overall, these results demonstrate that BBN-functionalized FGN-BBN selectively targets GRPR-expressing RM-1 cells, significantly enhancing their uptake through receptor-mediated endocytosis. Furthermore, the inherent FL of FGN-BBN enables real-time visualization of this targeted accumulation, establishing a foundation for precise GRPR-mediated imaging of metastasis PCa.

**Fig. 5:**
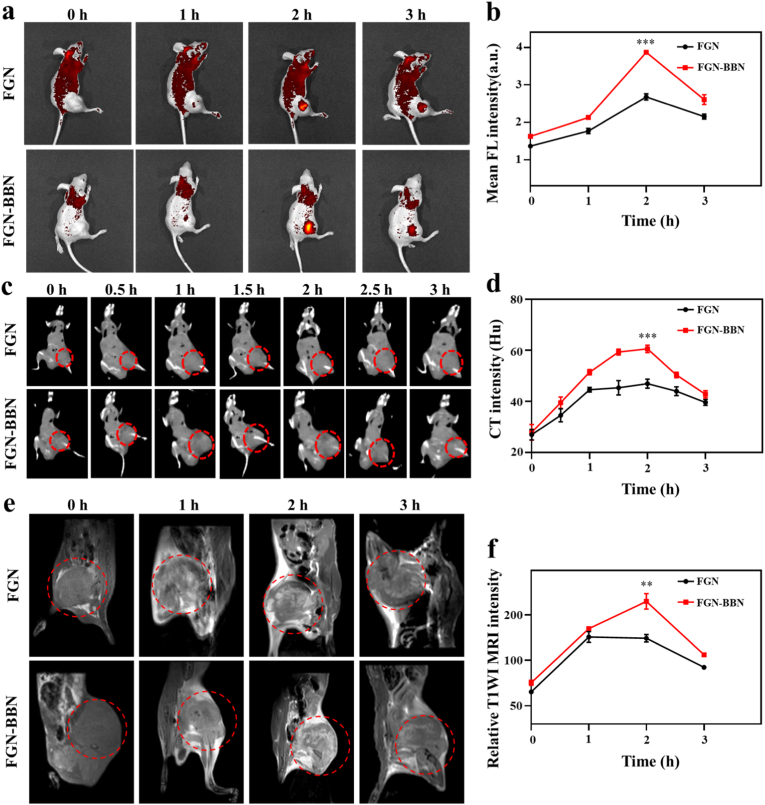
Multi-mode imaging (FL/CT/MR) of FGN-BBN in PCa bone metastasis. (a) In vivo FL images of different timepoints (0, 1, 2 and 3 h) following intravenous injection of FGN and FGN-BBN. (b) Quantitative analysis of FL intensity in tumor region. (c) CT images at different timepoints (0, 0.5, 1, 1.5, 2, 2.5, and 3 h) post-injection of FGN and FGN-BBN. (d) Quantitative analysis of CT intensity. (e) T1-weighted MR images at different timepoints (0, 1, 2 and 3 h) post-injection of FGN and FGN-BBN. (f) Quantitative analysis of MRI signal intensity in tumor site. ∗∗p < 0.01, ∗∗∗p < 0.001.

We next evaluated the GRPR-targeting specificity and in vivo multimodal imaging capabilities (FL/CT/MRI) of FGN-BBN in bone PCa metastasis model (Fig. 5). In vivo FL imaging and quantitative analysis demonstrated that, at 2 h post-intravenous administration, FGN-BBN exhibited a 1.44-fold increase in FL signal intensity compared to FGN (Fig. 5a and b). This enhancement suggests improved tumor accumulation of the FGN-BBN nanoplatform. Consistent with the FL imaging results, both CT and MRI signal also peaked at 2 h after intravenous injection of FGN and FGN-BBN (Fig. 5c and e). Compared to FGN, FGN-BBN showed a 1.28-fold increase in CT signal intensity and a 1.32-fold increase in MRI signal intensity, confirming enhanced imaging performance of FGN-BBN among all three modalities. To further validate the PCa-targeting capability of FGN-BBN, ex vivo FL imaging was performed on tumors and major organs harvested from mice bearing bone-metastatic PCa (Fig. S6 and S7). The FL signal in tumor tissue was markedly stronger in the FGN-BBN group compared to the FGN group, whereas no significant differences in FL intensity were observed among major organs between the two groups (Fig. S7). These results indicate that FGN-BBN selectively accumulates at the PCa site while maintaining low distribution in non-target tissues. The enhanced tumor localization is attributed to the overexpression of GRPR on PCa cells, which enables specific recognition and accumulation of the BBN-conjugated nanoplatform.

In conclusion, in GRPR-positive PCa bone metastasis model, FGN-BBN demonstrated excellent tumor-targeting capability and multimodal imaging performance (FL/CT/MRI). These results support its potential as a targeted imaging probe and provide a foundation for its application in imaging-guided therapy.

### 3.6 In vitro therapeutic ability and biocompatibility of FGN-BBN

**Fig. 6:**
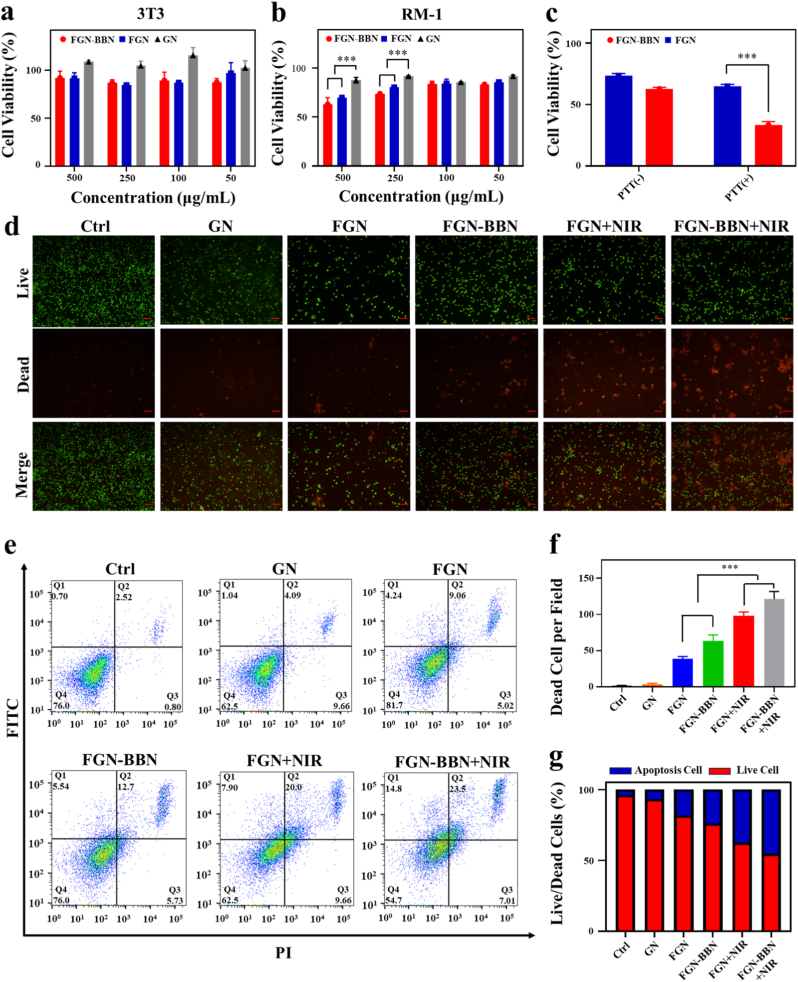
In vitro cytotoxicity and anti-tumor effect of FGN-BBN. (a) Viability of 3T3 fibroblast cells after 24 h co-incubation with GN, FGN, or FGN-BBN. (b) GN, FGN, and FGN-BBN induced cell death in PCa cells. (c) Synergistic cell-killing effect of FGN-BBN on RM-1 cells via PTT and ferroptosis under NIR irradiation. (d) Live/Dead cell staining of PCa cells following different treatments. Scale bar: 100 μm. (e) FCM analysis of apoptosis in RM-1 PCa cells treated with Ctrl, GN, FGN, FGN-BBN, FGN + NIR, and FGN-BBN + NIR. (f) Semi-quantitative analysis of live/dead cell staining. (g) Quantification of apoptotic cells from FCM results. ∗∗∗p < 0.001.

To evaluate the biocompatibility of FGN-BBN, a hemolysis assay was conducted to evaluate its compatibility with blood component (Fig. S5). In this assay, Triton X-100 was used as a positive control, whereas PBS served as the negative control. Hemolysis images showed that neither low nor high concentrations of FGN or FGN-BBN induced noticeable hemolytic reactions (Fig. S5a and S5b). This observation was further confirmed by quantitative absorbance measurements (Fig. S5c and S5d), indicating that the FGN-BBN nanoplatform does not induce hemolysis, thereby demonstrating excellent hemocompatibility—an essential requirement for intravenous biomedical application. To further investigate the biosafety of FGN-BBN, we used the NIH-3T3 fibroblast cell as a representative non-cancerous cell model. As shown in Fig. 6a, after co-incubation with different concentrations (50, 100, 250, and 500 μg/mL) of AuNDs (GN), FGN, or FGN-BBN, the viability of 3T3 cells remained above 80 % in all groups, demonstrating the low cytotoxicity of these gold-based nanomaterials. In contrast, the same concentrations of FGN and FGN-BBN exhibited cytotoxic effects against RM-1 PCa cells, especially at 250 and 500 μg/mL (Fig. 6b). These results suggest that FGN and FGN-BBN are selectively cytotoxic toward tumor cells while exhibiting minimal toxicity to normal cells, thereby confirming their tumor-targeted therapeutic potential. This selectivity can be attributed to the tumor microenvironment, where elevated levels of H_2_O_2_ are commonly present due to increased metabolic activity [32]. FGN-BBN exploits this difference by catalyzing the conversion of excess H_2_O_2_ into cytotoxic ·OH through Fenton reaction, thereby inducing tumor-specific oxidative damage.

Next, the in vitro therapeutic efficacy of FGN-BBN against PCa cells was evaluated (Fig. 6c). Both FGN and FGN-BBN exhibited PTT and CDT ability. Notably, due to the GRPR-targeting capability of FGN-BBN, it demonstrated a stronger cytotoxic effect than FGN alone. To further visualize cell viability and death status, live/dead cell staining was performed among different treatment groups (Fig. 6d and f). In the images, live cells are stained green, while dead cells are stained red. The FGN-BBN + NIR group showed the highest proportion of dead cells, followed by the FGN + NIR group, indicating that FGN-BBN-mediated PTT and PTT-enhanced CDT elicited potent cytotoxic effects in PCa cells. Compared with the GN and Ctrl groups, both FGN and FGN-BBN groups exhibited more extensive red FL, suggesting that the Fenton reaction contributes to PCa cell death in these treatment condition.

To further verify the status of cell death, apoptosis was quantified using flow cytometry (Fig. 6e and g). Compared with the Ctrl (4.0 %) and GN (6.8 %) groups, a significantly higher apoptotic rate was observed in the FGN (18.3 %) and FGN-BBN (24.0 %) groups. Upon NIR irradiation, the apoptotic rate further increased to 37.5 % in the FGN + NIR group and 45.3 % in the FGN-BBN + NIR group. These FCM results were consistent with the live/dead staining findings.

In addition, in vitro wound healing assay was performed to verify the anti-metastatic potential of FGN-BBN (Fig. S8). Compared with the Ctrl group, PCa cells treated with FGN, FGN-BBN, and FGN-BBN + NIR exhibited a noticeably reduced wound closure area after 24 and 48 h. This result suggests that FGN-BBN, particularly when combined with NIR irradiation, effectively suppresses the migratory potential of PCa.

In summary, these findings demonstrate that FGN-BBN exhibits excellent biocompatibility, with minimal toxicity toward normal cells. More importantly, through GRPR-targeted delivery, FGN-BBN selectively accumulates in PCa cells, enabling effective tumor recognition and cell killing. Upon NIR exposure, FGN-BBN acts as a potent PTT agent to induce localized hyperthermia, facilitating both direct tumor ablation and enhanced ROS production via photothermal-amplified CDT. Consequently, the synergistic integration of active targeting, PTT, and CDT not only leads to significant inhibition of PCa cell viability, but also effectively suppresses their migratory capacity. Furthermore, the observed excessive ROS accumulation and lipid peroxidation suggest a possible involvement of ferroptosis in the underlying mechanism of tumor cell death, which was further investigated in subsequent experiments.

### 3.7 Ferroptosis-inducing capability of the FGN-BBN nanoplatform

FGN-BBN was developed as a ferroptosis-inducing nanoplatform capable of amplifying intracellular oxidative stress, a key initiator of ferroptosis, which is characterized by iron-dependent lipid peroxidation mediated by excessive ROS. As a distinct form of regulated cell death, ferroptosis has attracted growing interest in cancer therapy owing to its unique molecular mechanisms and potent antitumor efficacy [33]. It is typically initiated by the inactivation of GPX4, depletion of intracellular cystine, or inhibition of the cystine/glutamate antiporter system Xc^−^ (SLC7A11), all of which disrupt redox balance and result in the toxic accumulation of ROS [34].

To effectively initiate ferroptosis in tumor cells, FGN-BBN promotes ROS accumulation through three synergistic mechanisms: (1). Fenton Reaction: FGN-BBN catalyzes the Fenton reaction, resulting in the generation of large amounts of ROS. (2). PTT Enhancement: The photothermal effect of FGN-BBN under NIR irradiation enhances the catalytic activity of the nanozyme, further increasing ROS production. (3). GSH Depletion: Released Fe^2+^ consumes intracellular GSH, impairs the antioxidant defense system, and inhibits ROS scavenging.

**Fig. 7:**
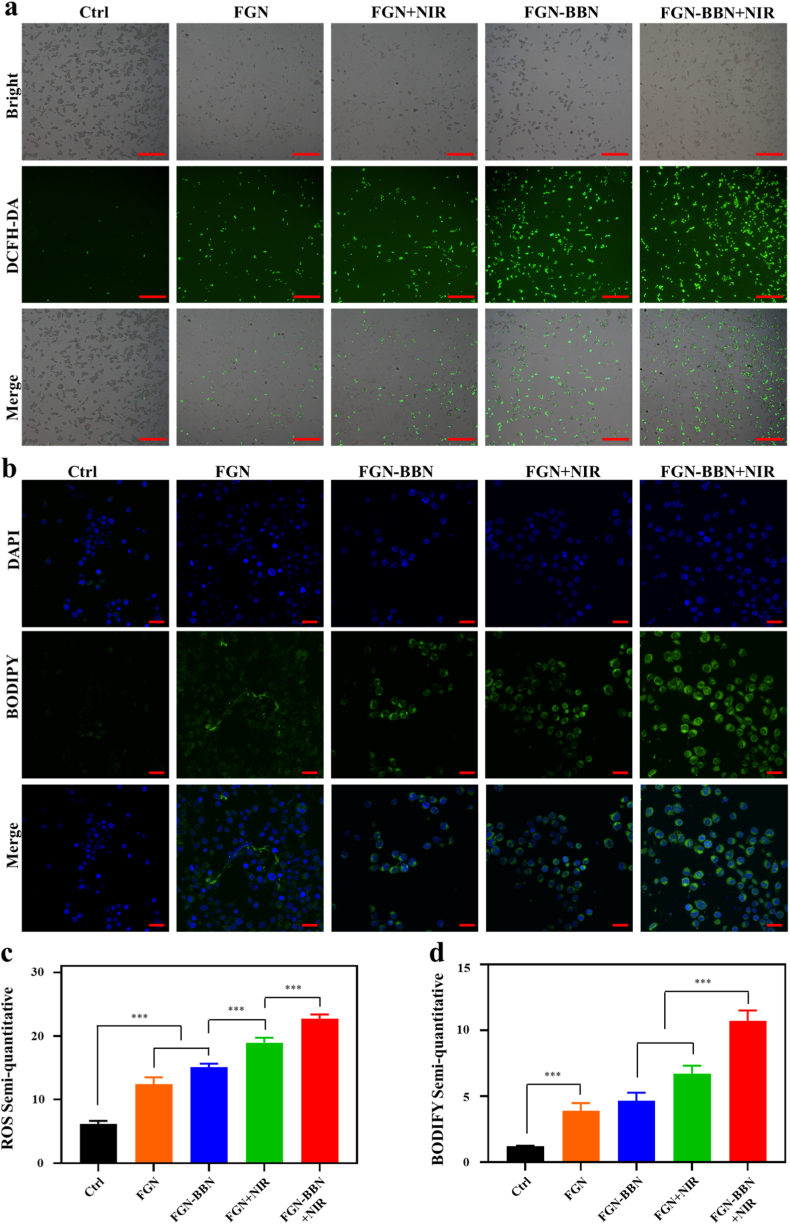
Detection of ROS generation and LPO after different treatments. (a) FL imaging of intracellular ROS levels in various treatment groups using DCFH-DA staining. Scale bar: 200 μm. (b) FL imaging of lipid peroxidation using BODIPY C11 staining. Scale bar: 20 μm. (c) Semi-quantitative analysis of ROS FL intensity. (d) Semi-quantitative analysis of LPO levels based on BODIPY FL. ∗∗∗p < 0.001.

Intracellular ROS levels were evaluated by using DCFH-DA staining (Fig. 7a and c). Among all groups, the FGN-BBN + NIR group exhibited the highest FL intensity, corresponding to a 3.68-fold increase in ROS levels compared to the control group. Furthermore, both FGN + NIR and FGN-BBN + NIR groups showed markedly higher ROS levels than their respective non-irradiated counterparts (FGN and FGN-BBN group), indicating that PTT stimulation significantly enhances ROS generation. This excessive accumulation of ROS cause DNA damage and lipid peroxidation, ultimately contributing to ferroptosis induction.

**Fig. 8:**
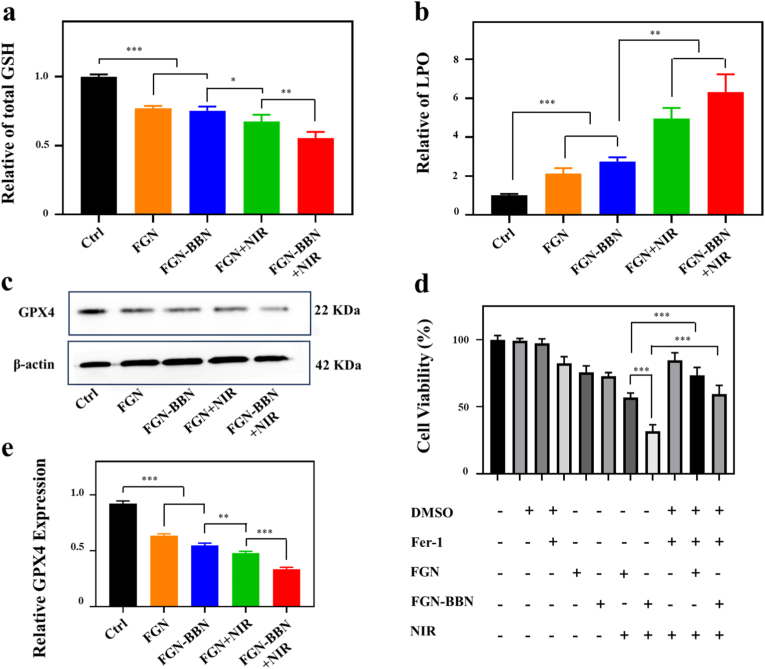
Assessment of ferroptosis-associated biomarkers. (a) Quantitative analysis of intracellular GSH levels following different treatments. (b) Quantitative analysis of LPO levels using MDA assay after different treatments. (c) Western blot analysis for GPX4 protein expression in various treatments group. (d) Cell viability assay with Fer-1 rescue experiment. ∗p < 0.05, ∗∗p < 0.01, ∗p < 0.001.

LPO, an important biomarker of ferroptosis, was assessed via BODIPY C11 staining (Fig. 7b and d) and MDA quantification (Fig. 8b). FL imaging revealed extensive LPO in the FGN-BBN + NIR group, indicating severe oxidative lipid damage (Fig. 7b). Correspondingly, quantitative MDA analysis revealed that the FGN-BBN + NIR (6.32-fold) and FGN-NIR (4.96-fold) groups exhibited significantly elevated MDA compared to control group. These results confirm that Fe^2+^ ions released from the FGN-BBN nanoplatform effectively promote LPO accumulation, thereby triggering ferroptosis in PCa cells [35]. Additionally, mild photothermal effects induced by NIR irradiation further enhanced ROS production, exacerbating LPO [36].

GSH is a key reducing agent that converts Fe^3+^ to Fe^2+^, but is also consumed during the Fenton reaction, thereby contributing to ROS amplification and ferroptosis progression.

To further validate the ferroptosis mechanism, intracellular GSH levels were evaluated in each treatment group (Fig. 8a). The GSH content in the FGN-BBN + NIR group decreased significantly (0.56-fold relative to control), which was lower than that in the FGN + NIR (0.68-fold), FGN (0.77-fold), and FGN-BBN (0.75-fold) groups. GSH serves as a key intracellular reducing component that facilitates the reduction of Fe^3+^ to Fe^2+^, but is also consumed during the Fenton reaction, thereby contributing to ROS amplification and ferroptosis progression.

In addition to GSH depletion, we investigated the expression of GPX4, a central regulator of ferroptosis. GPX4 is a selenoenzyme that catalyzes the reduction of lipid hydroperoxides and protects cells from oxidative damage. Suppression of GPX4 is frequently observed in ferroptosis related tumor death [37]. As shown in Fig. 8c, GPX4 expression was markedly downregulated in all treatment groups, with the most significant reduction observed in the FGN-BBN + NIR group (0.34-fold relative to control), followed by FGN + NIR (0.48-fold), FGN-BBN (0.55-fold), and FGN (0.64-fold). These results suggest under photothermal stimulation, FGN-BBN induces excessive ROS generation, depletes intracellular GSH, and downregulates GPX4 expression, thereby promoting ferroptosis in PCa cells.

To further verify the involvement of ferroptosis, rescue experiments were performed by using Fer-1, a classical ferroptosis inhibitor that suppresses lipid peroxidation by scavenging hydroperoxyl radicals [38]. As shown in Fig. 8d, Fer-1 treatment partially restored cell viability in FGN and FGN-BBN-treated groups, confirming that ferroptosis plays a dominant role in the observed cytotoxicity.

Taken together, these results demonstrate that FGN-BBN functions as an effective ferroptosis nano-inducer. Under NIR irradiation, it promotes ROS overproduction and disrupts the redox balance, leading to a “ROS storm” that drives ferroptosis-mediated tumor cell death.

### 3.8 FGN-BBN mediated in vivo PTT-ferroptosis therapy for bone metastatic PCa

**Fig. 9:**
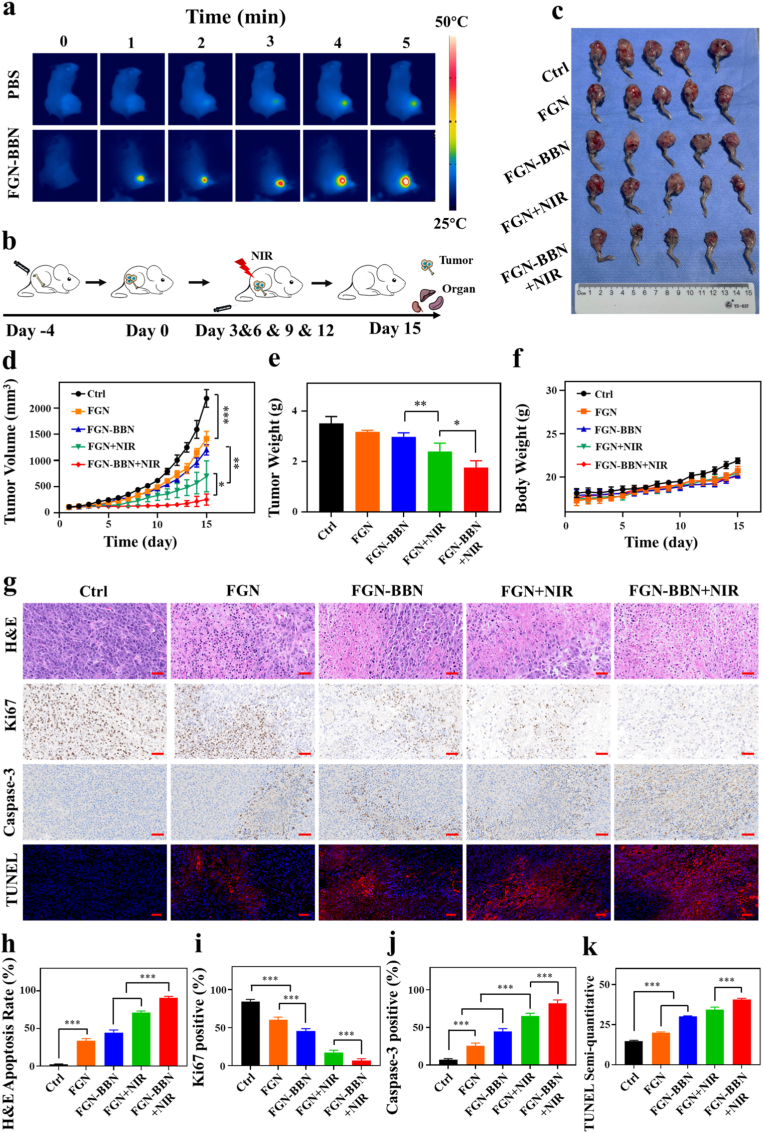
GRPR targeted photothermal with ferroptosis therapy effect and security evaluation of FGN-BBN in vivo. (a) Photothermal images of mice after intravenous injection of PBS or FGN-BBN under 808 nm laser irradiation at different time points. (b) Schematic diagram showing the establishment of PCa tibial metastasis model and nano-system treatment. (c) Tumor images for different groups. (d) Relative tumor volume curve. (e) Tumor weight after the anti-tumor experiment. (f) Body weight curve. (g) H&E-stained tumor tissue and immunohistology stained (Ki67, Caspase-3, and TUNEL). Scale bar: 100 μm (h) Semi-quantities analysis of apoptosis area, Ki67 positive percentage, Caspase-3 positive percentage and TUNEL positive percentage. Data are presented as mean ± SD (n = 5), ∗p < 0.05, ∗∗p < 0.01, ∗∗∗p < 0.001.

The in vivo therapeutic efficacy of FGN-BBN was systematically evaluated (Fig. 9). Initially, the in vivo photothermal conversion efficiency of the nanoplatform was evaluated. Following intravenous administration of FGN-BBN or PBS, mice were subjected to 808 nm laser irradiation (2.0 W/cm^2^), and real-time temperature changes at the tumor site were recorded using an infrared thermal imaging camera (Fig. 9a). With prolonged irradiation, the local tumor temperature in the FGN-BBN group rapidly increased, reaching approximately 50 °C, whereas only minimal temperature changes were observed in the PBS group. These results highlight the efficient photothermal conversion capability of FGN-BBN in vivo and its potential for tumor-targeted PTT.

Subsequently, in vivo therapeutic study was conducted using a PCa tibial metastasis model to further evaluate the antitumor efficacy of FGN-BBN (Fig. 9b). The mice with tibia PCa metastasis were randomly divided into 5 group: Ctrl, FGN, FGN-BBN, FGN + NIR and FGN-BBN + NIR. As demonstrated in previous targeting imaging experiments, FGN-BBN exhibited specific accumulation in GRPR-positive PCa. Based on this, NIR irradiation was applied to the tumor sites in the corresponding groups following systemic nanoplatform administration.

After four treatment cycles, tumors were excised for subsequent analysis. Representative images of the tumors from each group are shown in Fig. 9c. Consistent with the visual observations, the tumor growth curves showed that the FGN-BBN + NIR group exhibited the most significant tumor growth inhibition compared to all other groups (Fig. 9d). In addition, tumor weights were recorded post-treatment (Fig. 9e), and the FGN-BBN + NIR group showed significantly lower tumor weights than the FGN + NIR, FGN-BBN, FGN, and Ctrl groups. This significant therapeutic result is attributed to the synergistic action of FGN-BBN-mediated PTT and PTT-enhanced CDT, which together generate excessive ROS and induce ferroptosis in PCa bone metastasis.

To further validate the antitumor efficacy of FGN-BBN + NIR treatment, histological and IHC analyses were performed on excised tumor sections, including H&E staining, Caspase-3 and Ki67 IHC staining, as well as TUNEL immunofluorescence staining (Fig. 9g). H&E staining revealed that the FGN-BBN + NIR group exhibited the highest proportion of apoptotic tumor cells (90.76 ± 2.04 %), followed by FGN + NIR (71.2 ± 2.2 %), FGN-BBN (44.74 ± 3.34 %), FGN (33.64 ± 2.89 %), and the Control group (2.3 ± 0.72 %). Ki67, a widely used marker of cellular proliferation, showed the highest positive cell rate in the Control group (84.4 ± 2.7 %), indicating elevated proliferative activity. In contrast, the FGN-BBN + NIR group exhibited the lowest Ki67-positive rate (6.8 ± 2.28 %), confirming its strong inhibitory effect on tumor cell proliferation. To evaluate apoptosis induced by different treatments, tumor sections were analyzed for the expression of two key apoptotic markers: Caspase-3 and TUNEL. Compared with all other groups, the FGN-BBN + NIR group exhibited the highest TUNEL FL intensity (semi-quantitative value: 40.6 ± 0.8, p < 0.001) and the highest Caspase-3-positive cell rate (82.2 ± 4.49 %, p < 0.001). These results are consistent with the extensive necrosis observed in H&E staining. To evaluate the in vivo ferroptosis-inducing capability of FGN-BBN, IHC staining for GPX4 and 4-HNE was performed (Fig. S9). GPX4 is a key negative regulator of ferroptosis. As shown in Fig. S9a and S9c, GPX4 expression was markedly downregulated in the FGN, FGN-BBN, FGN + NIR, and FGN-BBN + NIR treatment groups, with the FGN-BBN + NIR group showing the lowest number of GPX4-positive cells among all groups. These findings further support the induction of ferroptosis in vivo. Lipid peroxidation is a hallmark of ferroptosis [39]. In this work, we employed an anti-4-HNE antibody to detect the accumulation of lipid peroxidation products in the tumor sections (Fig. S9a and S9b). The imaging and semi-quantitative analysis of positive density results showed a significant increase in 4-HNE positive staining in the FGN-BBN + NIR group compared with others. Meanwhile, Ctrl group exhibited the lowest 4-HNE positive density staining. This result also indicating FGN enhanced lipid peroxidation and caused ferroptosis in PCa bone metastasis.

Moreover, the in vivo biocompatibility of FGN-BBN was thoroughly evaluated. Body weight change is a key indicator of systemic toxicity. As shown in Fig. 9f, the body weight of mice in all treatment groups remained stable throughout the treatment period, indicating no significant adverse effects. Furthermore, H&E staining of major organs (heart, liver, spleen, lung, and kidney) revealed no noticeable histopathological abnormalities following FGN-BBN administration (Fig. S10). These findings further validate that the FGN-BBN-based PTT-ferroptosis therapy, synergistically enhanced by CDT and supported by precise GRPR-targeted imaging, achieves effective tumor suppression while maintaining excellent biocompatibility, with no evident systemic toxicity.

Overall, the FGN-BBN + NIR treatment exerts its therapeutic effect primarily through ferroptosis, with PTT acting as a synergistic enhancer. The localized hyperthermia generated by PTT amplifies the catalytic efficiency of CDT, leading to excessive ROS accumulation and efficient induction of ferroptosis in PCa cells. In addition to its therapeutic efficacy, FGN-BBN also demonstrated excellent in vivo biocompatibility, as evidenced by stable body weight in treated mice and the lack of histopathological abnormalities in major organs, indicating minimal systemic toxicity and favorable biosafety for potential clinical translation.

## 4 Conclusion

In summary, we successfully designed and synthesized a multifunctional nanoplatform FGN-BBN which serves as GRPR-targeting ferroptosis nano-inducer system with integrated diagnostic and therapeutic capabilities for bone-metastatic PCa. First, FGN-BBN nanoplatform enables multimodal imaging of GRPR-positive tumors. Owing to the inherent imaging capabilities of gold and iron, FGN-BBN nanoplatform supports FL, CT, and MR imaging, enabling accurate and comprehensive tumor visualization. Second, FGN-BBN functions as an effective ferroptosis nano-inducer. The Fe^2+^ component initiates Fenton reaction with intracellular H_2_O_2_ to generate abundant ROS and Fe^3+^, triggering ferroptosis. The resulting Fe^3+^ is subsequently reduced by GSH, establishing a self-sustaining Fe^2+^/Fe^3+^ redox cycle. Simultaneously, extensive GSH depletion further impairs the antioxidant defense system, leading to elevated ROS accumulation and enhanced ferroptosis. Importantly, the integration of PTT significantly enhances the ferroptosis effect. Au-mediated hyperthermia under NIR irradiation induces direct cytotoxicity and simultaneously boosts nanozyme activity and ROS production, ultimately enhancing ferroptosis. Therefore, FGN-BBN-mediated PTT combined with PTT-enhanced CDT effectively generates a large amount of ROS, which serves as a key mediator of ferroptosis. This synergistic mechanism greatly contributes to the observed tumor cell killing effect. By integrating GRPR-targeted multimodal imaging, PTT, CDT, and ferroptosis induction, the FGN-BBN nanoplatform enables precise tumor visualization and facilitates image-guided ferroptosis specifically for bone-metastatic PCa. Altogether, this work provides an effective and synergistic strategy for the targeted diagnosis and treatment of advanced bone-metastatic PCa.

## CRediT authorship contribution statement

**Liang He:** Writing – original draft, Visualization, Methodology, Formal analysis, Data curation. **Hao Liang:** Software, Methodology, Data curation. **Jixue Wang:** Software, Formal analysis, Data curation. **Annan Liu:** Software, Methodology, Data curation. **Lei Li:** Visualization, Software, Formal analysis. **Ji Lu:** Writing – review & editing, Supervision, Conceptualization. **Ze Wang:** Writing – review & editing, Supervision, Project administration, Methodology, Conceptualization. **Andrew K. Whittaker:** Supervision, Investigation. **Quan Lin:** Writing – review & editing, Supervision, Investigation, Conceptualization.

## Declaration of generative AI and AI-assisted technologies in the writing process

During the preparation of this work the authors used ChatGPT in order to text polishing. After using this service, the authors reviewed and edited the content as needed and take full responsibility for the content of the publication.

## Declaration of competing interest

The authors declare that they have no known competing financial interests or personal relationships that could have appeared to influence the work reported in this paper.

## Data Availability

No data was used for the research described in the article.
