# Supplementary material for: Multifunctional nanoplatform as nano-inducer of ferroptosis for targeted recognition and imaging-guided therapy of metastatic prostate cancer
Source: Mater Today Bio. 2025 Sep 18;35:102317. doi: 10.1016/j.mtbio.2025.102317 (PMC12510038; doi:10.1016/j.mtbio.2025.102317)
Supplement: Multimedia component 1 [file mmc1.docx]

**Multifunctional nanoplatform as nano-inducer of ferroptosis for targeted recognition and imaging-guided therapy of metastatic prostate cancer**

Liang He^1^, Hao Liang^2^, Jixue Wang^1^, Annan Liu^2^, Lei Li^2^, Ji Lu^1^*, Ze Wang^3,4^*, Andrew K. Whittaker^5^, Quan Lin^2^*

^1^ Department of Urology, the First Hospital of Jilin University, Changchun 130021, Jilin, China

E-mail: lu_ji@jlu.edu.cn

^2^ State Key Laboratory of Supramolecular Structure and Materials, College of Chemistry, Jilin University, Changchun, 130012, P. R. China

E-mail: [linquan@jlu.edu.cn](mailto:linquan@jlu.edu.cn)

^3^ Department of Hand Surgery, the Second Hospital of Jilin University, Changchun 130041, China

^4^ Joint International Research Laboratory of Ageing Active Strategy and Bionic Health in Northeast Asia of Ministry of Education, Changchun 130041, China

E-mail: [zewang@jlu.edu.cn](mailto:zewang@jlu.edu.cn)

^5^ Australian Institute for Bioengineering and Nanotechnology The University of Queensland

Brisbane, QLD 4072, Australia





**Fig. S1.** Zeta potential of FGN and FGN-BBN





**Figure S2.** High-resolution XPS spectra of S 2p





**Fig. S3.** Absorbance of MB after treatment with FGN-BBN and H₂O₂ under different pH conditions (5.0, 6.5, and 7.4).





**Fig. S4.** Stability of FGN-BBN assessed by FL intensity after 30-day storage under standard conditions.


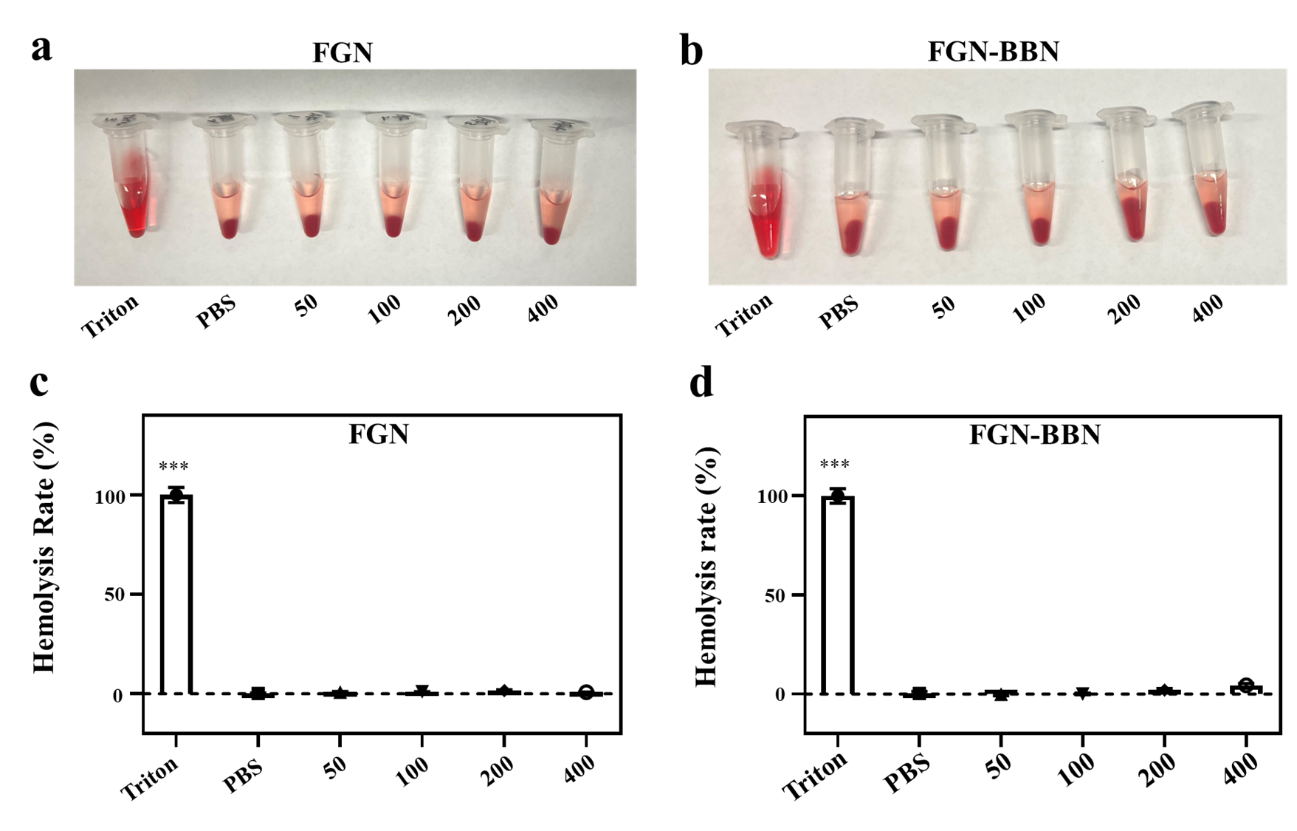


**Fig. S5.** Hemocompatibility evaluation of the FGN and FGN-BBN (a). Representative images of hemolysis at varying concentrations of FGN. (b) Representative images of hemolysis at varying concentrations of FGN-BBN. (c) Quantitative analysis of hemolysis percentages for FGN. (d) Quantitative analysis of hemolysis percentages for FGN-BBN.

**
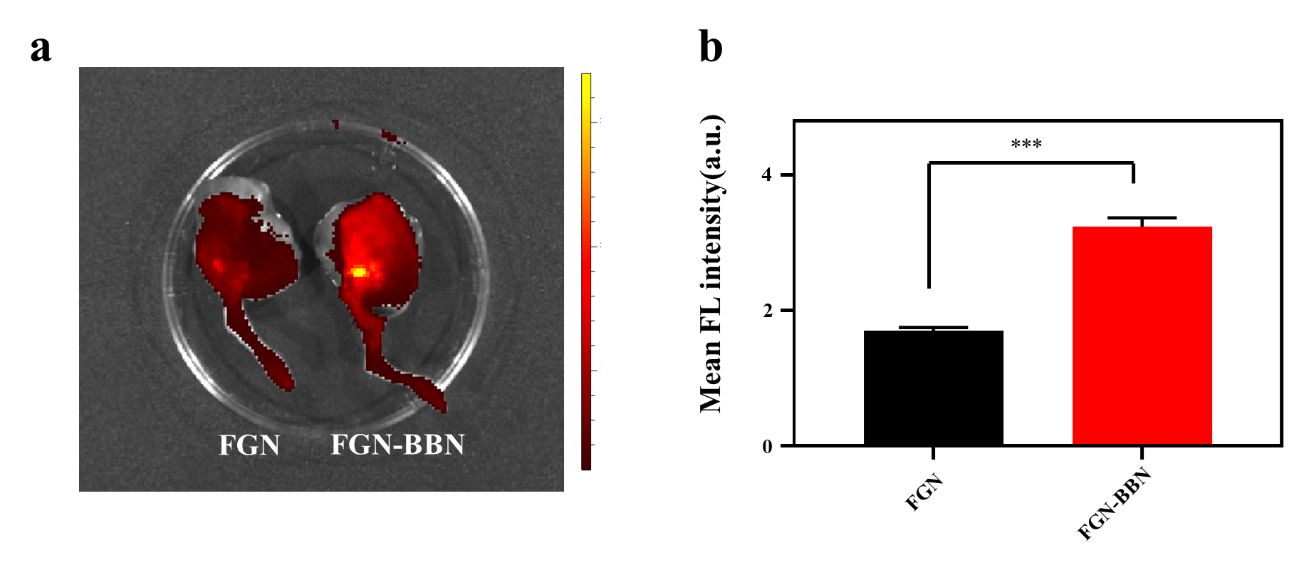
**

**Fig. S6.** Ex vivo FL imaging and signal quantification in the PCa bone metastasis model. (a) Representative ex vivo fluorescence images of tumor tissues harvested from mice treated with FGN or FGN-BBN. (b) Semi-quantitative analysis of FL intensities in excised tumor tissues from each group.

**
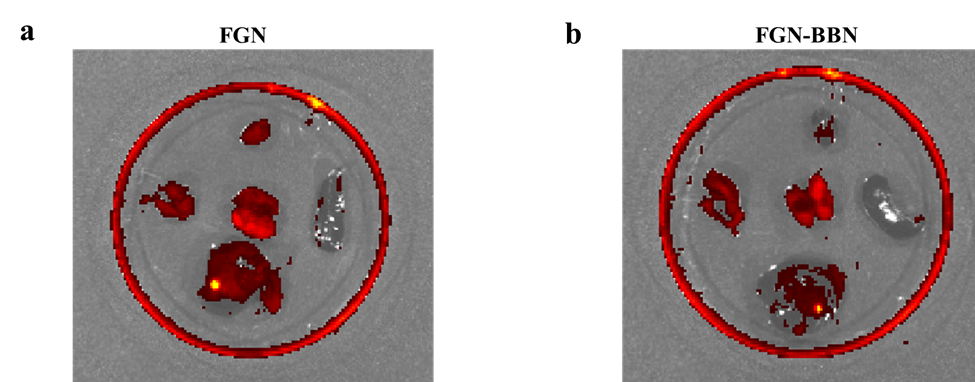
**

**Fig. S7.** Ex vivo FL imaging of major organs. (a) Representative FL images of major organs (heart, liver, spleen, lungs, kidneys, and tumor) excised from mice in the FGN group. (b). Representative FL images of major organs excised from mice in the FGN-BBN group.


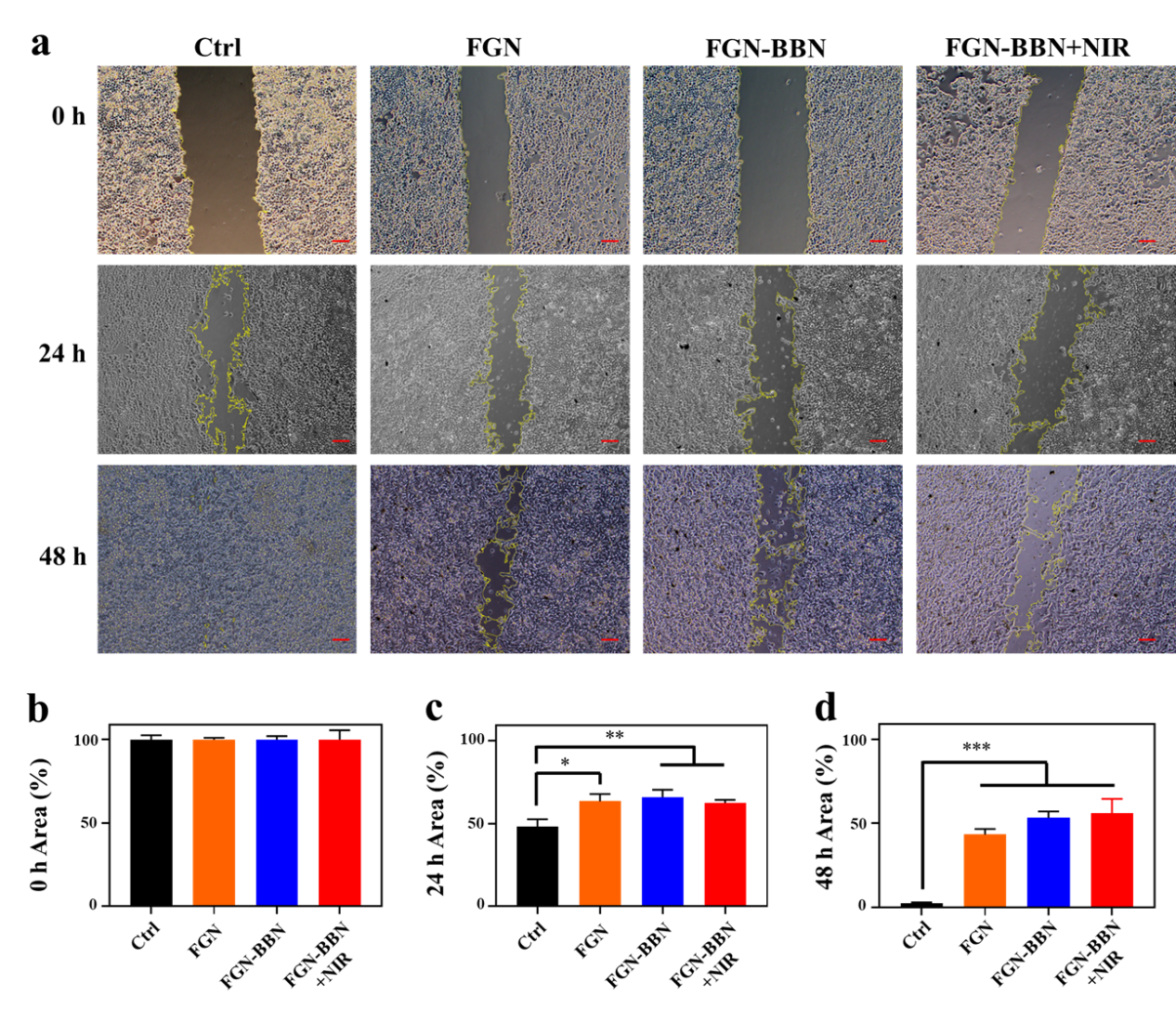


**Fig. S8.** Wound healing assay evaluating the migratory capacity of RM-1 cells under different treatments. (a). Representative bright-field images of cell migration at 0 h, 24 h and 48 h in the control, FGN, FGN-BBN, and FGN-BBN+NIR group. Yellow lines indicate the wound edges. Scale bar: 100 μm (b). Quantification of relative migration area in 0h. (c). Quantification of relative migration area in 24h. (d). Quantification of relative migration area in 48h. Data are presented as mean ± SD (n = 3). (* p<0.05, ** p<0.01, ***p<0.001).


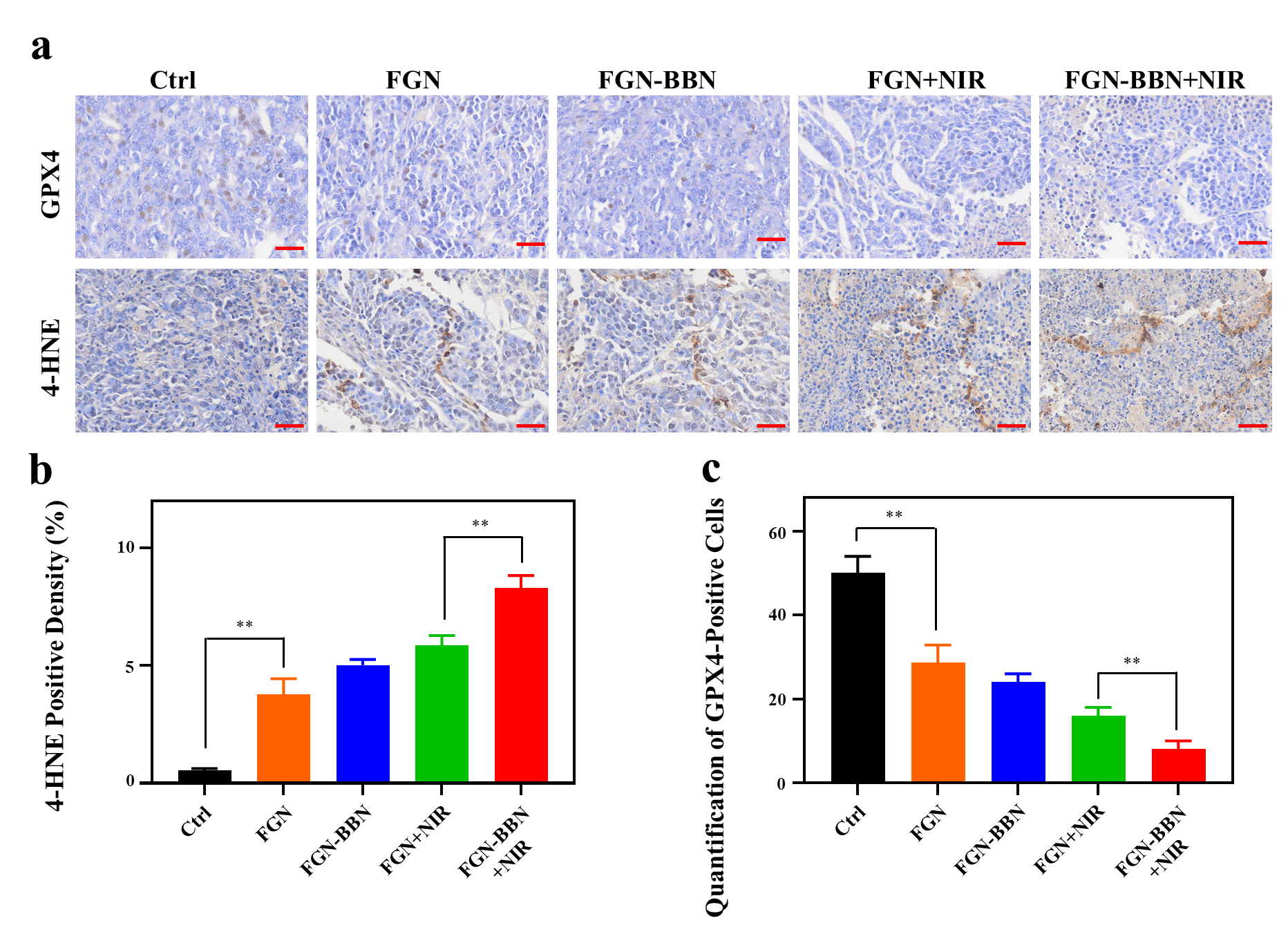


**Fig. S9.** Immunohistochemical analysis of 4-HNE and GPX4 expression in tumor tissues following different treatments. (a) Representative immunohistochemical staining of 4-HNE and GPX4 in tumor sections from each treatment group (Ctrl, FGN, FGN-BBN, FGN+NIR, and FGN-BBN+NIR). Scale bar: 50 μm. (b) Quantification of 4-HNE-positive staining area in each group, expressed as mean ± SD (n = 3). (c) Quantification of GPX4-positive cell number per field in each group, expressed as mean ± SD (n = 3). ** p<0.01.

**
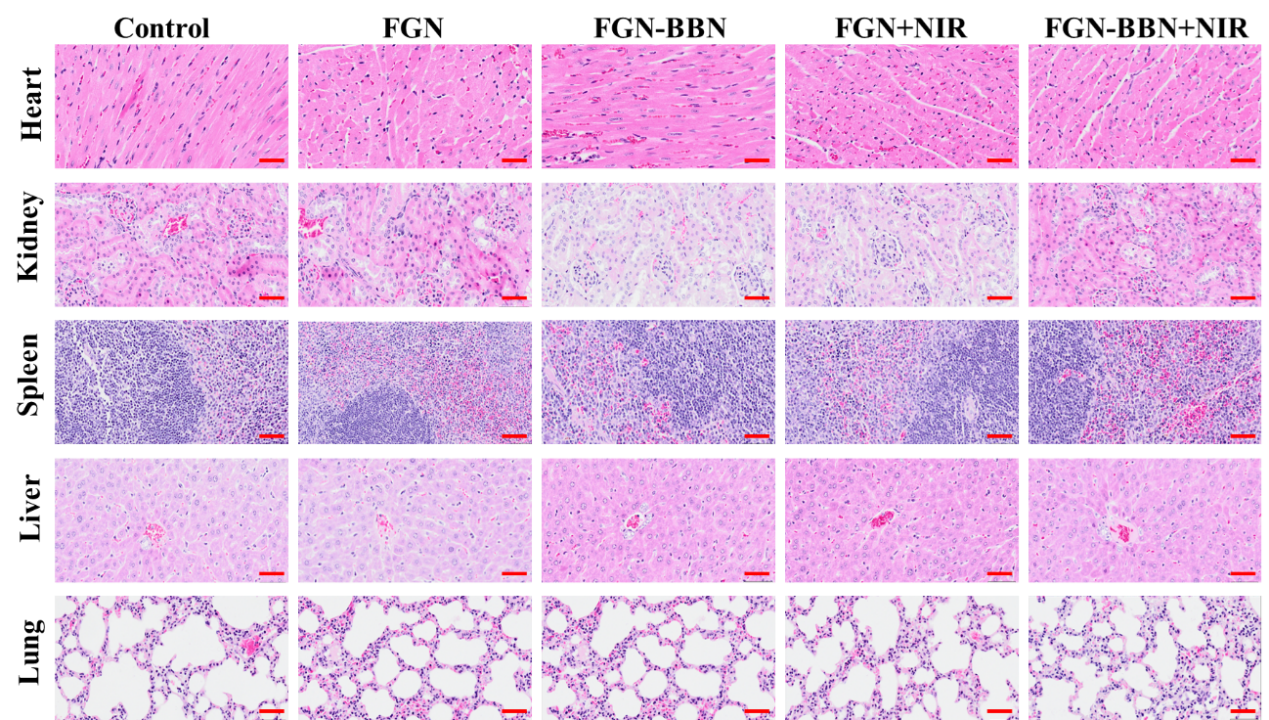
**

**Fig. S10**. H&E staining of major organ sections for different groups. Scale bar: 50 μm.
